# Eliminating interfacial O-involving degradation in Li-rich Mn-based cathodes for all-solid-state lithium batteries

**DOI:** 10.1126/sciadv.add5189

**Published:** 2022-11-25

**Authors:** Shuo Sun, Chen-Zi Zhao, Hong Yuan, Zhong-Heng Fu, Xiang Chen, Yang Lu, Yun-Fan Li, Jiang-Kui Hu, Juncai Dong, Jia-Qi Huang, Minggao Ouyang, Qiang Zhang

**Affiliations:** ^1^ Beijing Key Laboratory of Green Chemical Reaction Engineering and Technology, Department of Chemical Engineering, Tsinghua University, Beijing 100084, China.; ^2^ State Key Laboratory of Automotive Safety and Energy, School of Vehicle and Mobility, Tsinghua University, Beijing 100084, China.; ^3^ Advanced Research Institute for Multidisciplinary Science, Beijing Institute of Technology, Beijing 100081, China.; ^4^ Beijing Synchrotron Radiation Facility, Institute of High Energy Physics, Chinese Academy of Sciences, Beijing 100049, China.

## Abstract

In the pursuit of energy-dense all-solid-state lithium batteries (ASSBs), Li-rich Mn-based oxide (LRMO) cathodes provide an exciting path forward with unexpectedly high capacity, low cost, and excellent processibility. However, the cause for LRMO|solid electrolyte interfacial degradation remains a mystery, hindering the application of LRMO-based ASSBs. Here, we first reveal that the surface oxygen instability of LRMO is the driving force for interfacial degradation, which severely blocks the interfacial Li-ion transport and triggers fast battery failure. By replacing the charge compensation of surface oxygen with sulfite, the overoxidation and interfacial degradation can be effectively prevented, therefore achieving a high specific capacity (~248 mAh g^−1^, 1.1 mAh cm^−2^; ~225 mAh g^−1^, 2.9 mAh cm^−2^) and excellent long-term cycling stability of >300 cycles with 81.2% capacity retention at room temperature. These findings emphasize the importance of irreversible anion reactions in interfacial failure and provide fresh insights into constructing stable interfaces in LRMO-based ASSBs.

## Teaser

Li-rich Mn-based cathode interfaces were designed to inhibit the O-involving degradation for energy-dense lithium batteries.

## INTRODUCTION

High-energy density is the primary target for energy storage in the fast development of portable electronics and electric vehicles. Although continuous improvement has been achieved in lithium-ion batteries (LIBs) with organic liquid electrolytes (*1*), the growing safety problem arising from the flammable electrolytes and the desire to safely use the metal lithium anode have also spurred the development of all-solid-state lithium batteries (ASSBs) (*2*, *3*). By using solid electrolytes (SEs) and metal lithium, the ASSBs show great potential in facilitating a vertical leap in both energy density and safety (*4*, *5*). Furthermore, today’s SEs can deliver high ionic conductivity (10^−3^ S cm^−1^), which is comparable to that of commercial liquid electrolytes (*6*–*8*). Correspondingly, breaking the limitation of cathodic capacity is a determining factor toward high-energy density ASSBs.

Recently, the Li-rich cathode materials with the dual redox centers of both transition metal (TM) and oxygen redox reactions have gained much attention, which provides a new paradigm to deliver the capacity over 250 mAh g^−1^ (*9*–*11*). Beyond the conventional cathode materials, the special Li─O─Li configuration in Li-rich cathode materials triggers reversible anionic redox reactions and results in high capacity (*10*). Among various Li-rich cathodes, the Li-rich Mn-based oxide (LRMO) cathode material is a superior cathode candidate because of the advantages of outstanding sustainability apart from the superior electrochemical performances. The nontoxicity and highest crust abundance (~950 parts per million) of Mn element endow LRMO with ecofriendly and low-cost properties (*12*). Therefore, the ASSB composed of the high-capacity LRMO cathode and Li anode and the intrinsically safe inorganic SE holds promise for realizing the high-energy density, safety, and sustainability.

Nevertheless, unsaturated coordination of Mn─O bonds at the LRMO surface inhibits the charge transfer between the O and Mn ions and thus causes irreversible oxygen oxidation at high voltage (*13*, *14*), which inevitably deteriorates the LRMO|SE interface, resulting in sluggish ion transfer at the interface. Although tremendous strategies have been developed to improve the reversibility of anionic reactions for LRMO materials and remarkable progress has been achieved in LIBs (*14*, *15*), most of these strategies would induce inert components at the LRMO surface. Adopting these strategies in ASSBs inevitably blocks the Li^+^ transport at the LRMO|SE interface. Hence, the critical but less solved problem in LRMO-based ASSBs lies in the interfacial Li^+^ transport within solid-state LRMO cathode composite. Theoretically, eliminating O-involving degradation and generating favorable LRMO|SE interfacial ionic transport network at room temperature could effectively boost the electrochemical performance of ASSBs. Therefore, interfacial manipulation to achieve fast interfacial kinetics could revive research into LRMO cathodes in ASSBs and boost their electrochemical performance for practical applications.

**Fig. 1.**
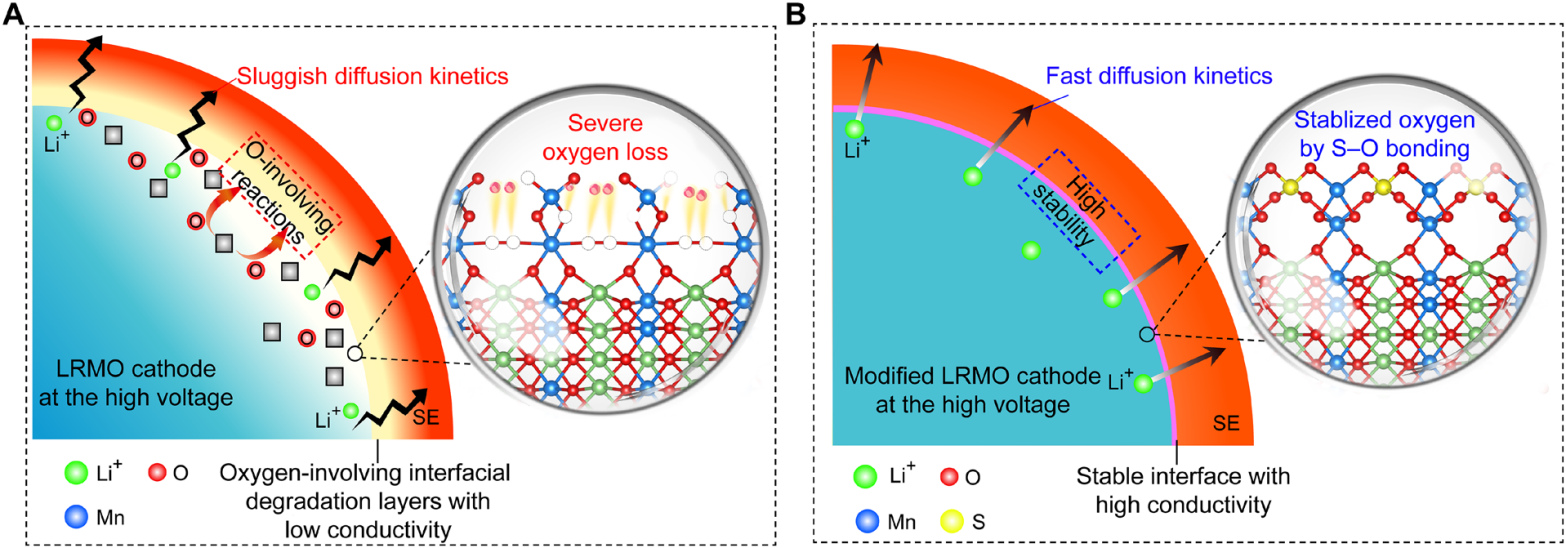
Interfacial manipulation by eliminating O-involving degradation. Schematic illustration of (**A**) the pristine LRMO with severe oxygen loss and oxygen-involving reactions at the interface and (**B**) modified LRMO by S─O bonding with the stable interface. SE, solid electrolyte.

In this contribution, an effective engineering strategy is proposed to tailor LRMO materials (Li_1.2_Mn_0.54_Co_0.13_Ni_0.13_O_2_) by bonding the sulfite (SO_3_^2−^) at the surface and forming high ionic conduction pathways with amorphous lithium sulfate simultaneously (Fig. 1). Replacing the weakly bonded surface oxygen with stable polyanion can prevent the overoxidation of surface oxygen through shifting the charge compensation of O^2−^ to SO_3_^2−^ in the process of charging. Because of the stable interface and favorable ionic transport network, the ASSB based on modified Li_1.2_Mn_0.54_Co_0.13_Ni_0.13_O_2_ (S-LRMO) cathode can deliver a high specific capacity (248 and 225 mAh g^−1^ at 1.1 and 2.9 mAh cm^−2^, respectively) and excellent long-term cycling stability of up to 4.6 V versus Li/Li^+^ in ASSBs (~81.2% capacity retention over 300 cycles at the rate of 1.0 C at room temperature). On the basis of in situ galvanostatic electrochemical impedance spectra (GEIS) and the distribution of relaxation time (DRT) technique, complemented by x-ray photoelectron spectroscopy (XPS), x-ray absorption spectroscopy (XAS), and time-of-flight secondary ion mass spectrometry (TOF-SIMS) characterizations and analyses, we first unravel the evolution of interfacial kinetics and interfacial chemistry according to the state of charge (SOC) in the solid-state LRMO cathode. It is demonstrated that the notable improvement in electrochemical performance lies in the stable S-LRMO|SE interface with favorable interfacial charge transfer during the cycling. This study emphasizes the importance of fast interfacial kinetics and provides a promising path for the rational design of LRMO materials to achieve high-energy density and long-term cycling stability of ASSBs.

## RESULTS

### Characterization of S-LRMO

**Fig. 2.**
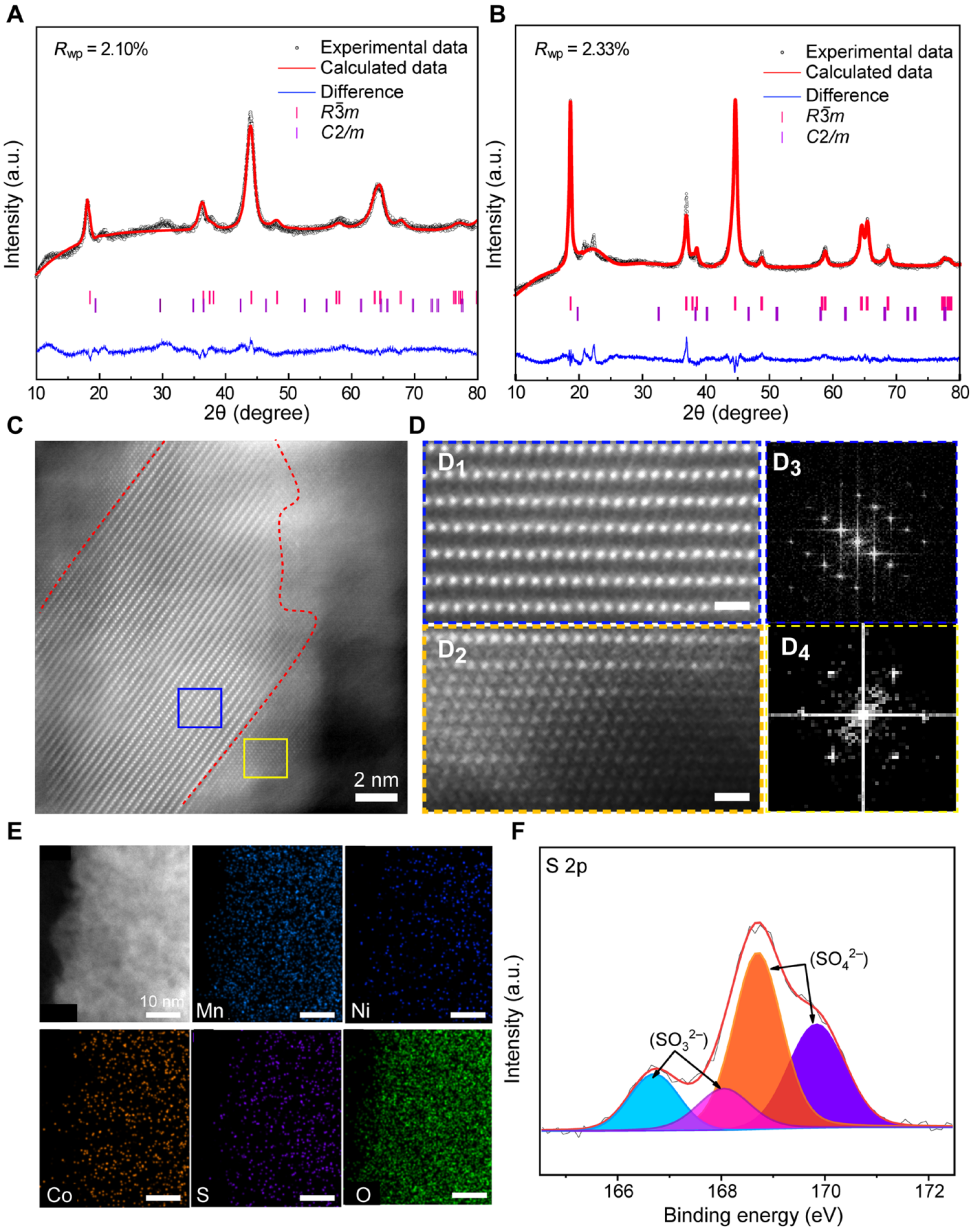
S-LRMO material characterizations. (**A** and **B**) XRD patterns with Rietveld fits for B-LRMO and S-LRMO, respectively. Refinements are conducted on the basis of Li_2_MnO_3_ and LiMO_2_ biphasic models. a.u., arbitrary units. (**C**) Representative high-angle annular dark-field scanning transmission electron microscopy (HAADF-STEM) image for the S-LRMO nanoparticle showing the layered rock salt intergrown structure. (**D**_1_ and **D**_2_) HAADF-STEM image for layered and rock salt Li_1.2_Mn_0.54_Co_0.13_Ni_0.13_O_2_ of the S-LRMO. Scale bars, 0.5 nm. (**D**_3_ and **D**_4_) Fast Fourier transformation (FFT) of the selected areas highlighted by blue and yellow box, respectively. (**E**) Energy-dispersive spectroscopy (EDS) mappings of Mn, Co, Ni, O, and S of S-LRMO. (**F**) S 2p spectra of S-LRMO.

The S-LRMO was prepared through a facile solid-state mechanochemistry of LRMO and Li_2_SO_4_ (fig. S1). For a fair comparison, the ball milling treatment of single LRMO (B-LRMO) is provided. The crystal structures of S-LRMO and B-LRMO are revealed by x-ray diffraction (XRD) patterns. All the reflections in Fig. 2 (A and B) can be well indexed to a two-phase composite, corresponding to a monoclinic unit cell (Li_2_MnO_3_, *C*2*/m*) and hexagonal unit cell (LiMO_2_, *M* = Mn, Ni, Co, *R*$\bar{3}$*m*) (*16*, *17*). Rietveld refinement of XRD for both B-LRMO– and S-LRMO–based *C*2*/m* and *R*$\bar{3}$*m* groups results in a good fit in diffraction peak (the weighted pattern factor, *R*_wp_ = 2.10 and 2.33%) (tables S1 to S4). No new Bragg peak or peak shift is observed, suggesting that the LRMO phases are well preserved and that Li_2_SO_4_ exists as an amorphous phase. However, the changed intensity ratio of diffraction peak (003)/(104) represents the degree of cations ordering in layered structure. Compared with pristine LRMO (fig. S2), the low-intensity ratio of B-LRMO and S-LRMO demonstrates the appearance of disordered rock salt phase (*18*, *19*). Further refinement of XRD patterns reveals ~11.5 and ~9.4% Li/Ni intermixing for B-LRMO and S-LRMO, respectively (tables S3 and S4).

The structure of S-LRMO is carefully probed at the atomic scale by transmission electron microscopy (TEM) and high-angle annular dark-field scanning TEM (HAADF-STEM). The morphologies, as shown in figs. S3 and S4, suggest the formation of intergrown domains of both S-LRMO and B-LRMO cathode materials. Figure S4 (C and D) reveals that nanosized layered phases (~5 nm) are intertwined with cationic disorder phases. Furthermore, an atomic-resolution HAADF-STEM of S-LRMO demonstrates the existence of disordered rock salt domains around layered domains (Fig. 2, C and D). The enlarged HAADF-STEM, as shown in Fig. 2 (D_1_ and D_2_), agrees well with the atomic arrangements of the ordered layered and disordered rock salt phases. Furthermore, the fast Fourier transform (FFT), as shown in Fig. 2 (D_3_ and D_4_), confirms the existence of a layered rock salt intergrown structure (*20*). In addition, the B-LRMO material also displays a similar intergrown structure (fig. S5). In contrast, the pristine LRMO without ball-milling treatment has the perfect layered structure (fig. S6). Note that such an intergrown structure exhibits a compatible boundary from the layered structure to the rock salt structure (fig. S7), which has been demonstrated to have nearly zero strain structural evolution during the electrochemical processes (*19*), in favor of the interfacial mechanical compatibility of cathode materials and SEs.

To unravel the surface composition evolution, the high-resolution energy-dispersive spectroscopy (EDS) mapping was carried out. As shown in Fig. 2E, the EDS mappings of Mn, Co, Ni, O, and S exhibit the uniform distribution of S element in the nanosized crystal bulk area. Meanwhile, as shown in fig. S8A, the amorphous structure highlighted by orange lines can be observed around disordered rock salt domains and layered domains. To further demonstrate the components of this amorphous structure at the atomic scale, the line profile analysis using STEM/EDS was applied along a linear path through the amorphous and crystalline regions. The spectra exhibit that S content increases in the amorphous region ranging from ~2 to ~4 nm, while Mn content decreases in this amorphous region, indicating such an amorphous region comprises Li_2_SO_4_ and Li_2_SO_3_ (fig. S8B). Furthermore, the core-level XPS spectra of B-LRMO and S-LRMO were used to accurately ensure their surface chemistry evolution. The O 1s core spectra of B-LRMO and S-LRMO demonstrate the existence of oxysulfide (fig. S9) (*21*). The S 2p XPS spectra of S-LRMO displays not only a pair of peaks located at 168.2 eV that is attributed to the SO_4_^2−^ but also a new pair of peaks at 166.3 eV that is from SO_3_^2−^ (Fig. 2F). Note that SO_3_^2−^ would not be generated by handling with the individual Li_2_SO_4_ powder under the same ball-milling conditions (fig. S9C). Moreover, it was also found that the Mn^3+^ ions were oxidized to the Mn^4+^ ions at the S-LRMO surface (fig. S10), whereas the evolution of Co and Ni valence was not observed, indicating that ball-milling treatment induces the chemical reactions between LRMO and Li_2_SO_4_ and generates charge transfer between Mn and S.

In addition, XAS was carried out to investigate the electron configurations and local structures of Mn ions in both S-LRMO and B-LRMO samples. The Mn x-ray absorption near-edge structure spectra (XANES) and *K*-edge extended x-ray absorption fine structure (EXAFS) of both B-LRMO and S-LRMO are shown in fig. S11. Compared to B-LRMO (fig. S11A), XANES Mn *K*-edge spectra of S-LRMO exhibits slightly higher energy, and thus S-LRMO have higher valence of Mn ions (*22*), which is consistent with the XPS results. In addition, FT of the Mn *K*-edge EXAFS was performed to demonstrate the local chemical environments of Mn (fig. S11B), in which two distinct shells around 1.4 and 2.4 Å can be observed, corresponding to Mn─O and Mn─Mn interactions, respectively (*23*). After ball-milling treatment with Li_2_SO_4_, the position of Mn─O and Mn─Mn barely changes. However, the intensity of S-LRMO is higher than that of B-LRMO, which can be ascribed to the high degree of disorder in B-LRMO. Given the XRD, TEM/STEM, XPS, and XAS results, it could be convincingly concluded that SO_3_^2−^ is bonded at the surface of S-LRMO and that unreacted amorphous Li_2_SO_4_ is located at the outer layer of SO_3_^2−^.

### Outstanding electrochemical performances of S-LRMO–based ASSBs

**Fig. 3.**
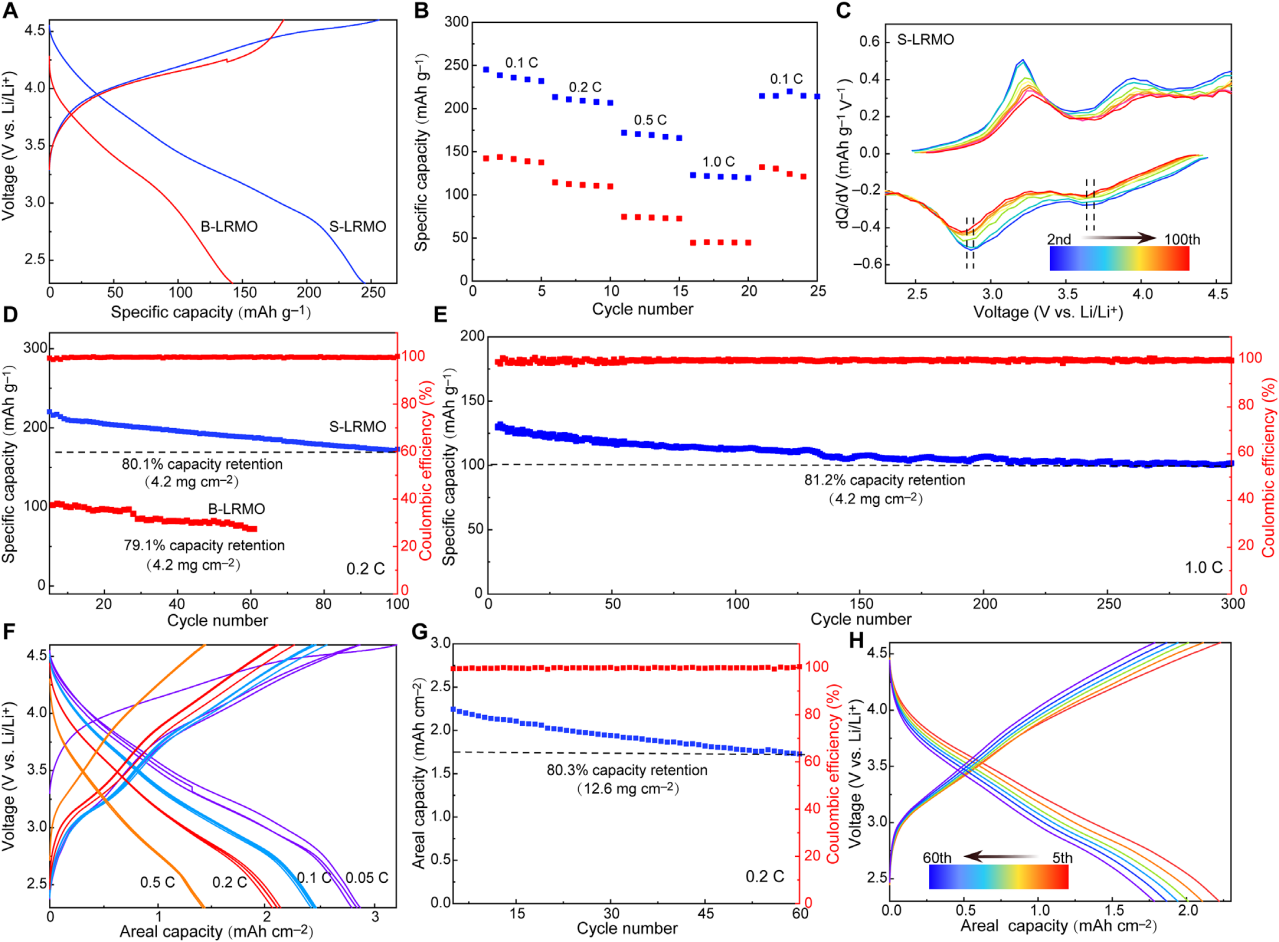
Electrochemical characterizations of ASSBs. (**A**) The first cycle voltage curves of B-LRMO and S-LRMO ASSBs. (**B**) Charge/discharge capacity at different current densities for ASSBs with B-LRMO and S-LRMO cathode-active materials. (**C**) dQ/dV profiles of the charge/discharge processes of S-LRMO ASSBs were collected from the 2nd to 100th cycle at 0.2 C. (**D**) Cycling performance comparison of B-LRMO and S-LRMO ASSBs at 0.2 C. (**E**) The long-term cycling of S-LRMO ASSB at the current density of 1.0 C. (**F**) Charge/discharge curves at different current densities for S-LRMO ASSBs with high loading. (**G**) Cycling performance of S-LRMO ASSBs with high loading at 0.2 C and (**H**) corresponding charge/discharge voltage curves.

ASSBs using B-LRMO and S-LRMO as the cathodes, dual-halogen SE Li_3_InCl_4.8_F_1.2_ (LICF) as the SE (*24*), and LiIn/In as the anode (see ASSBs structure in fig. S12) were constructed to evaluate the electrochemical performance of S-LRMO cathodes. LICF with high ionic conductivity (0.4 mS cm^−1^) provides favorable ionic pathways within the cathode composite and also acts as a separator (fig. S13). Meanwhile, the amorphous Li_2_SO_4_ has been demonstrated to have high ionic conductivity (*25*), resulting in the higher ionic conductivity of S-LRMO (7.5 × 10^−6^ mS cm^−1^) than that of B-LRMO (8.6 × 10^−7^ mS cm^−1^) (fig. S14, A to C). The first galvanostatic charge-discharge curves of B-LRMO and S-LRMO that were collected at a current density of 0.1 C with the voltage window of 2.3 to 4.6 V versus Li/Li^+^ are shown in Fig. 3A. The initial Coulombic efficiency (ICE) is 94% for S-LRMO, much higher than that of B-LRMO electrode (79%), demonstrating the improved reversibility of anionic redox. Figure 3B shows the calculated specific capacities of B-LRMO and S-LRMO as a function of the current density. A high specific capacity of 248 mAh g^−1^ is achieved for S-LRMO at the current density of 0.1 C. As the current density increases to 1.0 C, the S-LRMO still delivers a much higher specific capacity of 125 mAh g^−1^ (~50% capacity retention) than that of B-LRMO (44 mAh g^−1^ at 1.0 C, ~30% capacity retention) (fig. S15). This remarkably improved rate stability can be attributed to the eliminated O-involving degradation at the S-LRMO/LICF interface at the high voltage. The *dQ/dV* (Differential capacity over voltage) curves display the obvious suppression of the voltage fading in S-LRMO compared with that in B-LRMO (Fig. 3C and fig. S16). Long-term cycling behaviors of B-LRMO and S-LRMO at 0.2 C are shown in Fig. 3D. The S-LRMO ASSB shows minor capacity decay, retaining 80.1% capacity over 100 cycles at 0.2 C. In sharp contrast, the B-LRMO ASSB suffers from severe oxygen loss and causes severe S-LRMO|LICF interfacial degradation, thus exhibiting fast capacity decay with 80% capacity retention over only 60 cycles. Impressively, as the S-LRMO ASSB was subjected to prolonged cycling at 1.0 C (Fig. 3E), the corresponding capacity is 130 mAh g^−1^ at the initial cycle and maintains 81.2% capacity retention after 300 cycles, suggesting excellent interfacial stability between S-LRMO and LICF during the electrochemical processes. In addition, the ASSB based on S-LRMO exhibits excellent low-temperature performances and can retain 85, 73, and 61% of its room temperature capacity when cycled at −10°, −20°, and −30°C, respectively (figs. S17 to S19). Meanwhile, such a ASSB presents stable performance over 50 cycles at −30°C (96.2% capacity retention at 0.4 C) (fig. S19B). In contrast, the B-LRMO cathode delivers inferior low-temperature behaviors, which can be attributed to the O-involving degradation at the B-LRMO|LICF interface. In addition, the reversible capacity of S-LRMO in S-LRMO/SE/LTO (LTO = Li_4_Ti_5_O_12_) full cell is also exhibited in fig. S20 with an excellent cycling performance and a high discharge capacity of ~230 mAh g^−1^ at 0.1 C.

Although the above-mentioned ASSBs exhibit an outstanding electrochemical performance at the S-LRMO loading of 4.2 mg cm^−2^, high loading is more desirable to achieve the comparable areal capacity to the industry-level loading of ~3.0 mAh cm^−2^ (*26*). Figure 3F exhibits the ASSBs with high areal capacity (~2.9 mAh cm^−2^ at 0.05 C; 12.6 mg cm^−2^) cycled at room temperature. Moreover, the S-LRMO delivers good rate capability; 225 and 115 mAh g^−1^ can be achieved at 0.05 and 0.5 C, respectively, demonstrating fast ionic diffusion kinetics within S-LRMO cathode composites. S-LRMO ASSB with a high active material loading of 12.6 mg cm^−2^ can deliver outstanding long-term cycling stability of 80.3% over 60 cycles (Fig. 3, G and H).

### Interfacial transport properties

**Fig. 4.**
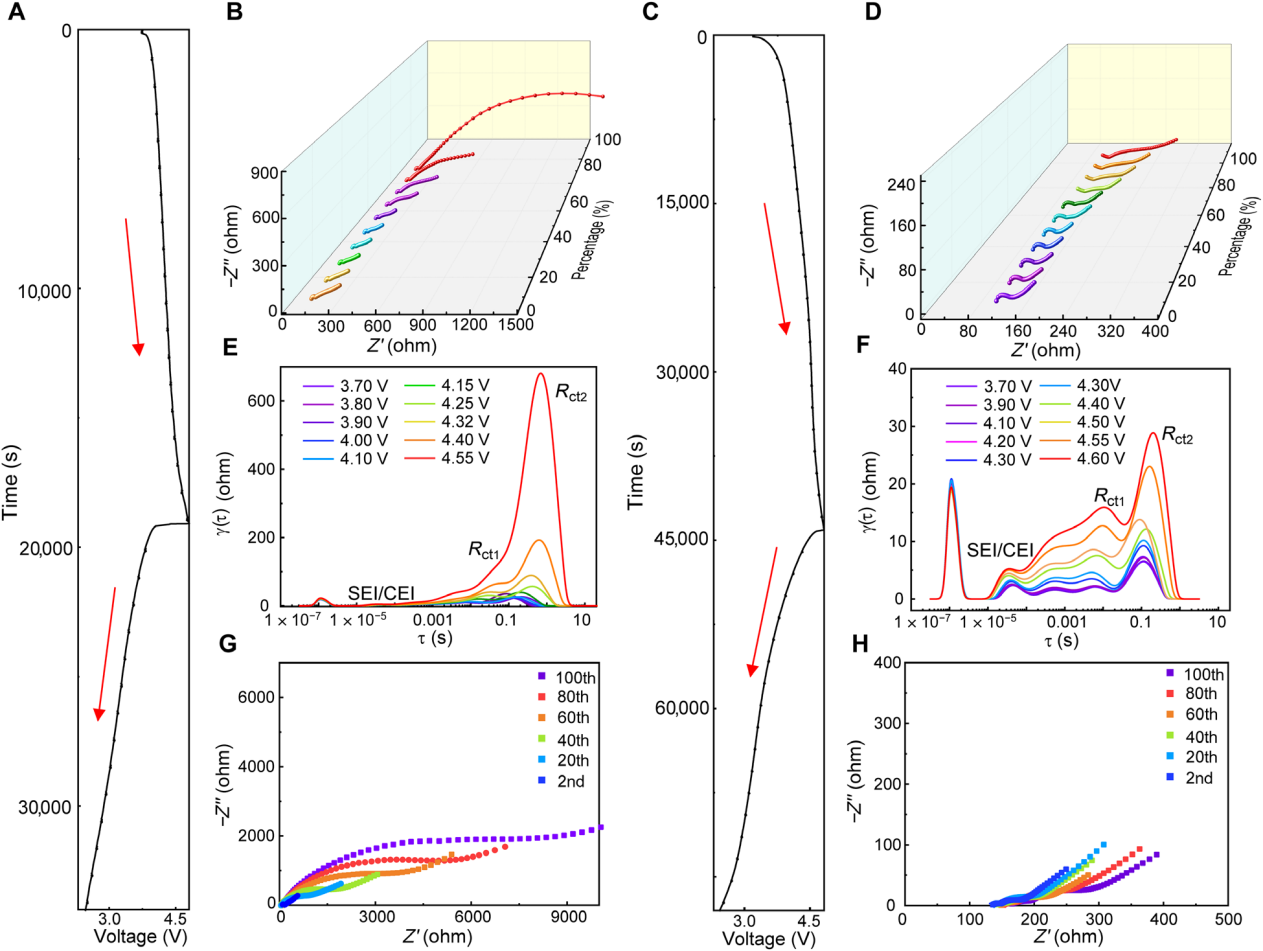
Interfacial Li^+^ kinetics evolutions in the cathode composites during cycling. (**A** to **D**) Interfacial impedance evolution during the first cycle of the B-LRMO and S-LRMO ASSBs cycled between 2.3 and 4.6 V versus Li/Li^+^. The corresponding (A and C) charging/discharging curves and (B and D) impedance spectra collected during initial charging of the B-LRMO and S-LRMO ASSBs are shown. (**E** and **F**) DRT profile transformation from GEIS of B-LRMO and S-LRMO ASSBs, respectively. (**G** and **H**) Nyquist plots of B-LRMO and S-LRMO ASSBs cycled between 2.3 and 4.6 V versus Li/Li^+^ for 100 cycles at full discharge.

In ASSBs, the marked changes of electrochemical behavior are originated from interfacial Li^+^ kinetics evolution, which can be uncovered by using the in situ GEIS measurements in combination with DRT analysis. Figure 4 (A to D) exhibits the impedance evolution of the ASSB based on the B-LRMO and S-LRMO at different cutoff voltages. Compared to the Li|SE interface, the cathode|SE interfacial resistance obviously dominates the whole resistance (fig. S21). Specifically, as shown in Fig. 4B, for B-LRMO ASSBs, the change of interfacial resistances with elevating SOC is mainly in the mid-/low-frequency region, expanding from approximately 50 to 2000 ohms as open-circuit voltage (OCV) increases to 4.6 V versus Li/Li^+^ (*27*). In sharp contrast, the S-LRMO ASSB exhibits a small change in the mid-/low-frequency region, only increasing from 20 to 100 ohms (Fig. 4D), which is far less than the change in B-LRMO ASSBs, suggesting the stable and fast interfacial diffusion kinetics in the S-LRMO cathode composites during the electrochemical process.

To accurately distinguish the evolution of the cathodes, DRT technology is used. Note that DRT is independent of experience-based models, which can display the time domain–based spectra based on a mathematical transformation of the frequency domain–based Nyquist plots (*4*, *28*). The achieved relaxation-based function γ(τ) can express the specialized electrochemical process by the formation of peaks at the specific relaxation time. The impedance value is represented by the peak areas. Correspondingly, the DRT technology can decouple the resistance evolution in cathode composites during the Li-ion intercalation/deintercalation processes. Specifically, the peak at 10^−6^ s has been demonstrated to correspond to the grain boundary of SEs (*4*). The impedance of the anode can be ruled out by the DRT result of symmetrical Li/Li cells, in which one peak located at 10^−5^ to 10^−4^ s exhibits a stable state originating from the formation of solid electrolyte interphase (SEI) (fig. S21B). Thus, the other peak located at 10^−3^ to 10^−1^ represents the charge transfer process (*R*_ct_).

Moreover, the EIS spectra in Fig. 4 and figs. S22 to S24 are converted into DRT functions, respectively. For B-LRMO–based ASSBs, as shown in Fig. 4E, four peaks located at 10^−5^ to 10^−4^, 10^−3^ to 10^−2^, 10^−2^ to 10^−1^, and 10^−1^ to 1 s represent the electrochemical processes of the cathode and anode sides. On basis of the DRT analysis of symmetrical Li/Li and S-LRMO/S-LRMO cells (figs. S21B and 22B), it can be confirmed that the peaks located at 10^−2^ to 10^−1^ and 10^−1^ to 10^0^ s (*R*_ct1_ and *R*_ct2_, respectively) are attributed to the resistance of the charge transfer at the cathode|SE interface. In addition, the peaks at 10^−5^ to 10^−4^ s is ascribed to SEI/CEI (cathode electrolyte interphase) (*4*). Note that the LRMO is made up of two phases, and thus two observed peaks corresponding to *R*_ct1_ and *R*_ct2_ in DRT analysis are reasonable. As shown in Fig. 4E, *R*_ct1_ and *R*_ct2_ at the B-LRMO|LICF interface are observed at the beginning of the charging and increase sharply over 4.2 V, whereas those at the S-LRMO|LICF interface increase slowly even at the high voltage of 4.6 V (Fig. 4F), suggesting the greatly suppressed interfacial degradation and fast interfacial diffusion kinetics in the S-LRMO cathode composite. During the discharging process (figs. S23 and S24), the interface in B-LRMO cathode composites still maintains high resistance, indicating the irreversible and severe interfacial degradation at the high voltage. In addition, Nyquist plots for B-LRMO (2.3 to 4.6 V versus Li/Li^+^; Fig. 4G) ASSBs collected at the fully discharged state cycled at 0.2 C exhibit tremendous internal resistance after 100 cycles, increasing to ~7000 ohms. However, the corresponding Nyquist plots of S-LRMO ASSBs (Fig. 4H) only increase to ~300 ohms. This remarkably stable impedance of S-LRMO ASSBs demonstrates the benefit of interfacial stability to interfacial diffusion kinetics.

To further quantify the electrode kinetics, galvanostatic intermittent titration techniques (GITTs) were conducted. The chemical diffusion coefficient of lithium (*D*_Li^+^_) were calculated in B-LRMO and S-LRMO electrode during the charging process. As shown in fig. S25A, it can be observed that the *D*_Li^+^_ of the S-LRMO is similar to the B-LRMO below the 4.0 V in the range of 10^−9^ to 10^−11^ cm^2^ s^−1^. Once the voltage goes beyond 4.0 V, the *D*_Li^+^_ of the S-LRMO (10^−10^ to 10^−11^ cm^2^ s^−1^) is one to two orders of magnitude higher than that of the B-LRMO (10^−11^ to 10^−13^ cm^2^ s^−1^). It indicates that the diffusion of Li^+^ is much slower in B-LRMO than that in S-LRMO, especially at the high voltage, which can be ascribed to the irreversible phase transition induced by the oxygen loss. Meanwhile, the overpotential η increases up to ~400 mV in B-LRMO, whereas the η in S-LRMO is ~200 mV (fig. S25, B and C). The rapidly decreasing chemical diffusion coefficient of lithium and growing polarization in B-LRMO at high voltage are highly related to the interfacial degradation at the high voltage (*29*), which is consistent with the GEIS and DRT results.

### Interfacial structural evolution and chemical mechanisms

**Fig. 5.**
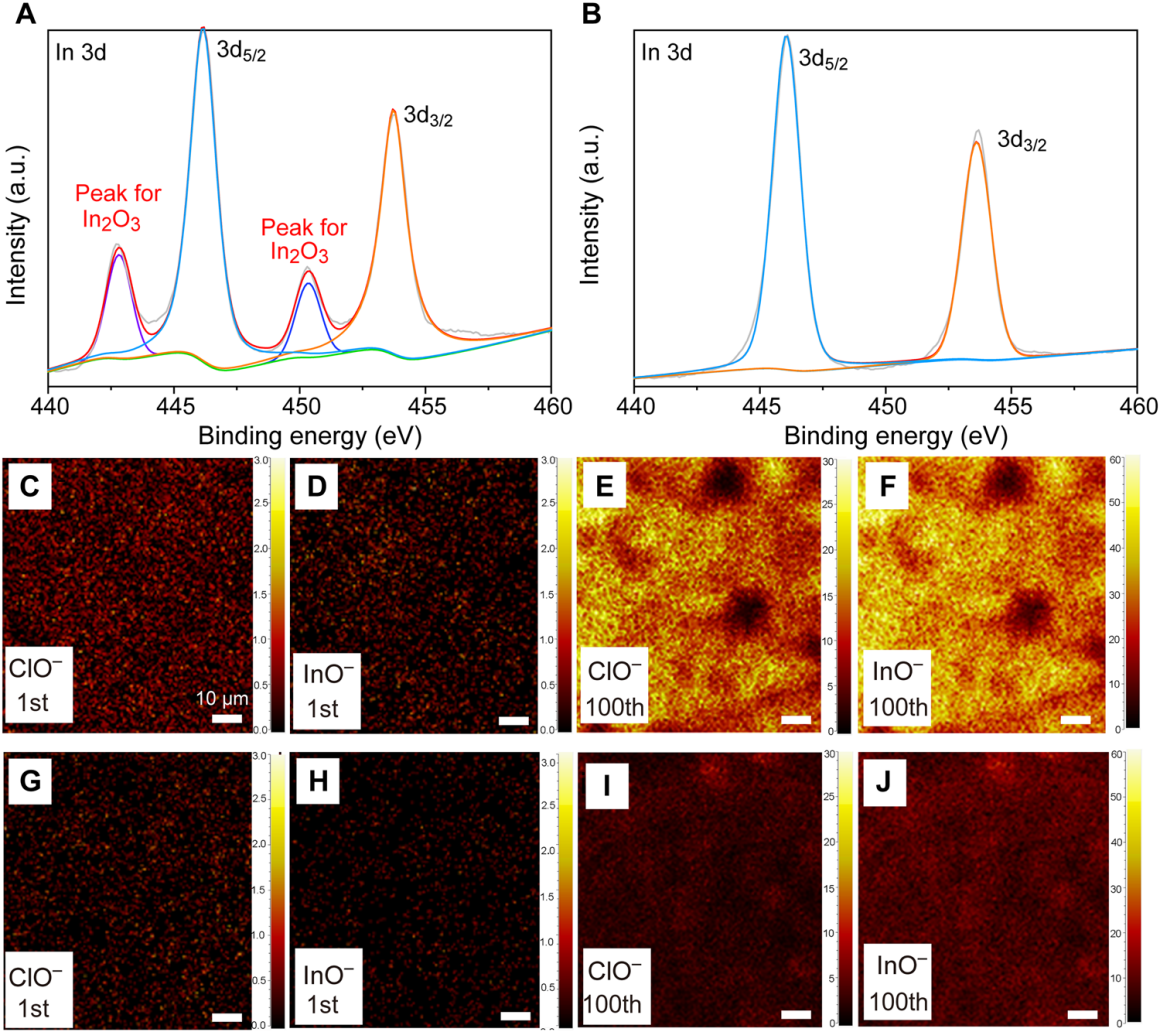
Interfacial chemical evolutions between LRMO and LICF during cycling. (**A** and **B**) In 3d XPS spectra were collected at 4.6 V versus Li/Li^+^ for the B-LRMO and S-LRMO cathode composites, respectively. (**C** to **F**) Time-of-flight secondary ion mass spectrometry (TOF-SIMS) analysis results of ClO^−^ and InO^−^ in B-LRMO cathode composites with cutoff potentials of 4.6 V versus Li/Li^+^ (first and 100th cycle). (**G** to **J**) TOF-SIMS analysis results of ClO^−^ and InO^−^ in S-LRMO cathode composites with cutoff potentials of 4.6 V versus Li/Li^+^ (1st and 100th cycle).

Ex situ XPS and TOF-SIMS analysis were further used to evaluate the interfacial stability. In 3d_3/2_ and In 3d_5/2_ peaks are located at the 453.8 and 446.2 eV, respectively (*30*, *31*). It can be observed that the two extra peaks appear in the In 3d spectra of the LRMO cathode composite at a constant voltage of 4.6 V after the first charge, which is attributed to the generation of In_2_O_3_ in LICF, demonstrating the O-involving degradation at the B-LRMO|LICF interface (Fig. 5A). In contrast, apart from the In 3d_3/2_ and In 3d_5/2_ peaks from LICF, In_2_O_3_ peaks are not observed in the S-LRMO cathode composite (Fig. 5B). Complementary chemical insight into interfacial degradation was further obtained by the TOF-SIMS measurement, which is highly sensitive to the fragments with high ionization probabilities and has been demonstrated to efficiently characterize the SE degradation at the cathode side (*32*). The B-LRMO cathode composites collected at the high cutoff potential (4.6 V versus Li/Li^+^) after first and 100th cycles were compared to those of S-LRMO cathode composites. The appearance of InO^−^ and ClO^−^ fragments could be the evidence of the interfacial O-involving degradation, which derives from the phase transitions of LRMO and the caused decomposition of halogen SEs (*33*). Both signals in B-LRMO cathode composites show more pronounced signal intensities than those in S-LRMO during the charging (Fig. 5, C, D, G, and H). In addition, the intensity of InO^−^ and ClO^−^ signals in B-LRMO cathode composites significantly increases after 100 cycles, revealing the continuously growing degradation layers at the interface. As expected, the S-LRMO cathode composites with highly reversible anionic reactions display the slightly increased signal intensities of InO^−^ and ClO^−^ even after 100 cycles (Fig. 5, E, F, I, and J). It is also observed that LiF^−^ is formed during the initial charging process and the SE is uniformly distributed around LRMO during the electrochemical processes, as shown in figs. S26 to S28. In addition, the cross-sectional SEM along with the EDS mapping of the cycled SE cathode composites displayed in fig. S29 suggests the superior interfacial mechanical compatibility, which can be attributed to the layered rock salt intergrown structure with low strain during the electrochemical process (*19*).

**Fig. 6.**
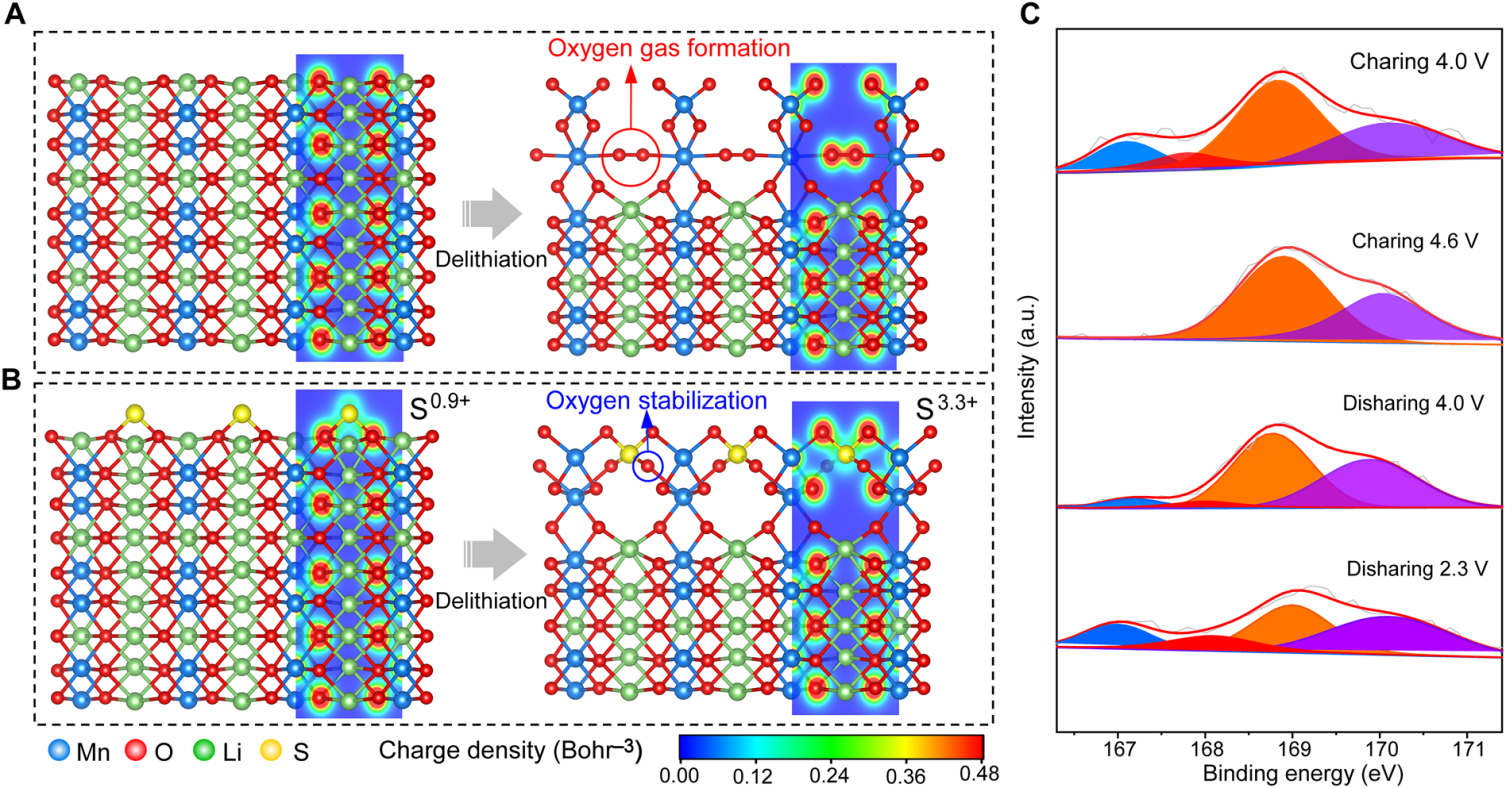
Strong interfacial polyanionic bondings to stabilize surface oxygen. (**A**) Atomic structure and corresponding charge density distribution of the (010) surface with the formation of O─O dimers in the delithiation process. (**B**) Atomic structure and corresponding charge density distribution of the (010) surface with the stabilized surface oxygen by generating S─O polyanions during the delithiation. (**C**) Ex situ S 2p spectra for the S-LRMO cathodes during the first charging/discharging process.

The stability of surface oxygen is the key to reversible anionic reactions. Therefore, we carried out the density functional theory (DFT) calculations to reveal the surface oxygen evolution. Previous reports have demonstrated that the anionic redox reactions in bulks are generally reversible because of kinetic prohibition for the migration of formed peroxide dimer or trapped oxygen molecules (*11*, *34*, *35*). Here, the observed low-index Li_2_MnO_3_ (010) surface (Fig. 2C) was investigated. Several Li-ion layers at the surface were removed to simulate the delithiation state. The surface O ions spontaneously formed O─O dimers during the delithiation process (Fig. 6A). The bond length of the O─O dimers is 1.24 Å, close to that in O_2_ molecules (1.21 Å). The Bader charge analysis was further performed to probe the charge evolution during the delithiation process (*36*). The O valence at the surface changed from −1.16 to −0.11, which can be observed in the charge density evolution around O─O dimers, indicating the formation of oxygen gas molecules (*14*). When the S─O bond was introduced on the (010) surface, no O─O dimers were observed during the delithiation process. Note that in Fig. 6B of the charge density distribution, the O electron cloud polarizes toward S, indicating a polyanionic bonding nature. The S atoms in SO_3_^2−^ participated in the charge compensation, i.e., a significant change of the S valence from +0.9 at the lithiated state to +3.3 at the delithiated state, which is demonstrated by the obvious charge depletion around S.

In addition, we conducted ex situ XPS and Raman spectroscopy measurements to verify this charge compensation process of sulfite from the perspective of experiments (Fig. 6C). This result indicates that SO_3_^2−^ bonding with the LRMO could be oxidized to the SO_4_^2−^during the charging process, which is in accordance with the calculation results. The SO_3_^2−^/SO_4_^2−^ redox reaction is reversible during the discharge process. The lost SO_3_^2−^ signal in the charging process reappears when the S-LRMO is discharged below 4.0 V. These reversible reactions of SO_3_^2−^/SO_4_^2−^ can be derived from the strong interactions between SO_3_^2−^/SO_4_^2−^ and Mn ions. Note that the electron could be extracted from Mn ions to the S center via the inductive effect of SO_4_^2−^ (*21*, *37*). The ex situ Raman spectroscopy, as shown in fig. S30, further convinces the highly reversible reactions of SO_3_^2−^/SO_4_^2−^ (*38*, *39*). Moreover, the evolution of O 1s spectra for S-LRMO and B-LRMO during the electrochemical process (fig. S31) also demonstrates the role of SO_3_^2−^ bonding in improving the structural stability. Specifically, the O 1s spectrum of the S-LRMO cathode barely changes when the electrode is oxidized to 4.3 V, indicating no significant oxygen redox reaction at this voltage in the first cycle. However, the O 1s spectrum undergoes markedly change when the electrode is oxidized to 4.6 V, namely, the appearance of new oxide ions located at ∼530.5 eV with a lower electron density than O^2−^ ions. Such a peak can be ascribed to the O^*n*–^ (*n* < 2) (*40*), implying that partially oxidized O^2−^ ions exist in the crystal of the S-LRMO electrode. In the following discharge process, the reduced intensity of O^*n*–^ demonstrates the reduction reactions of O^*n*–^. It disappears when the electrode is discharged to 3.0 V and the shape of spectrum converts back to the pristine S-LRMO, suggesting the high reversibility of the oxygen redox reactions in the S-LRMO electrode. In sharp contrast, a portion of the O^*n*–^ peak remains when the B-LRMO electrode is discharged to 2.0 V, indicating the existence of severely irreversible reactions for oxygen ions in the B-LRMO electrode (fig. S32).

To track the structural change and chemical behavior of Mn ions in the S-LRMO cathode during charge and discharge process, we carry out ex situ XAS measurement (*41*, *42*). Figure S33 (A and B) exhibits the ex situ Mn *K*-edge XANES spectra during the first charge and discharge process. When the S-LRMO cathode is delithiated from the OCV to 4.6 V, the spectrum of Mn exhibits barely edge shift, indicating that the redox couple is anionic oxygen at high voltage. During lithiation of S-LRMO from 4.6 to 3.0 V, Mn spectrum is mostly unchanged, indicating that Mn redox is not responsible for the reactions in this region. Upon further lithiation to 2.0 V, Mn spectrum obviously shifts to lower energy, suggesting the reduction of Mn ions to contribute to the capacity. This reaction process of Mn ions is also demonstrated by the ex situ XPS of Mn 3s spectra (fig. S34). In addition, FT of the Mn *K*-edge EXAFS was performed to demonstrate the local chemical environments of Mn. In comparison to the pristine state (fig. S33C), the intensity of Mn─O/Mn─Mn peaks decreases when the S-LRMO electrode is oxidized to 4.6 V due to an increase in disorder induced by the charge compensation of oxygen (*43*). During the discharge process, the signal of Mn─O/Mn─Mn increases after discharge to 3.0 V, suggesting the high reversibility of the local structure of S-LRMO. However, the intensity of Mn─O/Mn─Mn decreases significantly when delithiated to 2.0 V, which derives from the following reduction process of Mn ions. Therefore, the introduction of SO_3_^2−^ shifts the redox center from the O^2−^/O^−^ to the SO_3_^2−^/SO_4_^2−^, which can prevent the surface oxygen dimerization and effectively stabilize S-LRMO|LICF interface during the delithiation process (*44*), resulting in superior electrochemical performance of the ASSB based on high-loading S-LRMO.

## DISCUSSION

In summary, it is demonstrated that a simple balling-milling treatment, which is perfectly compatible with the state-of-the-art LRMO ASSBs, can stabilize the surface oxygen of S-LRMO, inhibit S-LRMO|LICF interfacial degradation, and improve interfacial Li-ion transfer kinetics for maximum performance. The underlying mechanisms are unraveled by advanced TOF-SIMS, XAS, and in situ GEIS cooperating with DRT analysis. In B-LRMO cathode composites, unstable surface oxygen structure triggers the oxygen migration and thus leads to the failure of the B-LRMO|LICF interface, resulting in markedly increased interfacial resistance. The S-LRMO with highly stable oxygen structure inhibits the oxygen loss by the charge compensation of bonded sulfite (SO_3_^2−^) and thus endows the S-LRMO|LICF interface with high stability. Correspondingly, the ASSB based on S-LRMO cathode composite exhibits greatly reduced capacity loss in the initial cycle (ICE = 94%) and delivers superior long-term cycling stability up to 4.6 V versus Li/Li^+^ in ASSBs (approximately 81.2% capacity retention over 300 cycles at the current density of 1.0 C). It is desirable that this finding concerning rationally designing interfacial chemistry in LRMO cathode composites can open up an avenue to develop high-safety LRMO ASSBs with high-energy density.

## MATERIALS AND METHODS

### Experimental section

#### Materials synthesis

##### Synthesis of pristine LRMO materials

LRMO was prepared through coprecipitation as reported previously (*45*). Specifically, a 1.0 M solution was produced by dissolving stoichiometric NiSO_4_·6H_2_O, CoSO_4_·H_2_O, and MnSO_4_·H_2_O, with a Ni/Co/Mn molar ratio of 1.3:1.3:5.4 into distilled water. A 2.0 M NaOH solution was further added to the obtained solution and stirred vigorously under the N_2_ atmosphere. To maintain the pH value of 11, the proper NH_4_OH is added.

Subsequently, the precipitate could be achieved by filtering, washing, and drying at 80°C for 24 hours in a vacuum oven. Last, the pristine LRMO was prepared by mixing hydroxide precursor with stoichiometric Li_2_CO_3_ via ball milling and further heating to 900°C for 12 hours in air.

##### Synthesis of B-LRMO and S-LRMO materials

The B-LRMO and S-LRMO materials were prepared by mechanically milling 0 and 20% Li_2_SO_4_ with pristine LRMO, respectively. In detail, these mixtures were added into a zirconia pot with a powder-to-ball mass ratio of 1:10. The milling process was conducted in a planetary apparatus (MITR, QM-QX-0.4 L) at 500 revolutions per minute (rpm) for 60 hours. All processes were performed in Ar atmosphere.

##### Synthesis of LICF SE

LICF was synthesized from InCl_3_ (>99.999%; Strem Chemicals), LiCl (≥99.99%; Sigma-Aldrich), and InF_3_ (anhydrous, ≥96%; Alfa Aesar). The stoichiometric amount of raw materials was mechanochemically milled at 500 rpm for 20 hours with a 10:1 mass ratio of ball and powder. Subsequently, the mixed products were transferred into quartz tubes and annealed at 260°C for 5 hours in the vacuum, and the LICF material was achieved after cooling down to room temperature.

#### Material characterization

The SEM was performed using JSM 7401F to characterize the morphology of materials and cross sections. XRD (Bruker D8 diffractometer) and Raman (Renishaw inVia) were applied to characterize cathode materials structure. Corresponding rietveld refinement was conducted using General Structure Analysis System (GSAS) II. The surface/interface chemistry was analyzed by XPS (Kratos Analytical, Axis Supra+) with an Al Kα radiation, and all electrodes collected at different voltages were etched by Ar for 10 s with an etch voltage of 5 kV to ensure the bulk phenomenon. TOF-SIMS (5 to 100; IONTOF GmbH) is also carried out to characterize the surface/interface chemistry. The atomic structure was characterized by the TEM (JEOL, 2100Plus) performed operated at 180 kV, and the HADDF-STEM was conducted using a Cs-corrected STEM (FEI Titan Cubed Themis G2 300) operated at 300 kV. Mn *K*-edge XAS measurements were performed at beamline 1W1B of the Advanced Photon Source at Beijing Synchrotron Radiation Facility (Beijing, China).

#### Electrochemical measurements

ASSBs using the LRMO cathode active material in combination with the LICF SE and the In/InLi anode were assembled in an argon-filled glovebox. A thin layer of Li_6_PS_5_Cl with high ionic conductivity was added between the anode and LICF to prevent the reduction of LICF. Li_6_PS_5_Cl SE is from Zhejiang Ningbo Linengxin New Materials Corp. Ltd. First, 40 mg of Li_6_PS_5_Cl was transferred into a polyaryletheretherketone (PEEK) cylinder and pressed at 360 Mpa for 2 min. Then, 80 mg of LICF was pressed on the Li_6_PS_5_Cl with the same condition. The cathode composite mixture was obtained by mixing cathode active materials with LICF and carbon nanotube with a weight ratio of 60:40:5. On the LICF side, ~6 or 18 mg of cathode composites were pressed at 360 MPa for 3 min. On the other side, an indium foil (with a diameter of 10 mm diameter and a thickness of 100 μm) was pressed, and then a thin Li foil (with a diameter of 10 mm and a thickness of 30 μm) was attached to the indium foil. Last, this cell was fixed into a stainless steel plate casing with an applied pressure of ∼20 MPa. LRMO/SE/LTO all-solid-state batteries with a diameter of 10 mm were assembled using LTO as the anode, Li_6_PS_5_Cl/LICF as the SE, and S-LRMO/LICF mixture as the cathode.

Galvanostatic cycling of the ASSB was operated the voltage ranges from 2.3 to 4.6 V versus Li/Li^+^ at different rates using LAND battery test system (Wuhan LAND Electronic Co. Ltd., China). The in situ EIS is tested by Solartron EnergyLab XM, in which the frequency ranges from 1 MHz to 0.1 Hz, and the amplitude is 10 mV. The repetitive GEIS measurements are conducted during the charging and discharging process after an equal interval time of 0.5 hours. In addition, the DRT analyses are transited by the MATLAB Graphical user interface (GUI) toolbox that was developed by Ciucci’s research team (*46*). The ionic conductivity was tested by EIS. Specifically, ~50 mg of SE was spread over the stainless steel rods and then pressed at 350 Mpa for 2 min. EIS was operated under the same condition.

#### Calculation of diffusion coefficient

The chemical diffusion is characterized by the GITT, in which the relaxations in cathode materials are originated from the Li^+^ diffusion. The diffusion coefficient is calculated by the following equations (Eqs. 1 and 2) (*21*, *29*, *47*)

$$D_{\text{GITT}}=\frac{4}{\pi \tau }{\left(\frac{n_{m}V_{m}}{S}\right)}^{2}{\left(\frac{∆E_{s}}{∆E_{t}}\right)}^{2}$$ (1)

$$\tau \ll \frac{L^{2}}{D_{\text{GITT}}}$$ (2)

where τ represents the relaxation time, *n*_m_ is the mole value, *V*_m_ represents the mole volume, *S* represents the contact area of cathode/SE, ∆*Es* represents the voltage response under the pulse current, and ∆*Et* is the voltage change through the galvanostatic discharge.

#### DFT calculation

DFT calculations were performed to reveal the stabilization mechanism of layered MoO_3_ induced by the pinning effect using the Vienna Ab initio Simulation Package (*48*). The projector augmented wave method was used to describe the ion-electron interactions (*49*). The Perdew-Burke-Ernzerhof version of the generalized gradient approximation (GGA) was adopted for the exchange correlation energy (*50*). A kinetic energy cutoff of 520 eV was used for the plane wave expansion of the valence electron wave functions. A dense Γ-centered Monkhorst-Pack *k*-point mesh with a sampling density of 0.04 Å^−1^, a total energy of 10^−6^ eV per cell, and a force of 10^−2^ eV/Å were adopted for the convergence criterion during structural optimization. Because of the layer structure, van der Waals (vdW) density functional of optB86b-vdW functional was performed during structural optimization. Considering the strong electron correction effect in TM oxides, electronic structure calculations were performed by a GGA plus Hubbard *U* (GGA + *U*). A *U* value of 4.5 eV was set for the d electrons in Mn atoms. A Li_2_MnO_3_ (010) surface model was constructed on the basis of the experimental XRD data. A vacuum layer of 15 Å was set to avoid the artificial interlayer interaction due to the periodic boundary condition.
