# Supplementary material for: Eliminating interfacial O-involving degradation in Li-rich Mn-based cathodes for all-solid-state lithium batteries
Source: Sci Adv. 2022 Nov 25;8(47):eadd5189. doi: 10.1126/sciadv.add5189 (PMC9699669; doi:10.1126/sciadv.add5189)
Supplement: Supplementary file 1 — Figs. S1 to S34 Tables S1 to S4 [file sciadv.add5189_sm.pdf]

Science Advances
AAAS

Supplementary Materials for

Eliminating interfacial O-involving degradation in Li-rich Mn-based cathodes for all-solid-state lithium batteries

Shuo Sun *et al.*

Corresponding author: Chen-Zi Zhao, zcz@mail.tsinghua.edu.cn;
Qiang Zhang, zhang-qiang@mails.tsinghua.edu.cn

Sci. Adv. **8**, eadd5189 (2022)
DOI: 10.1126/sciadv.add5189

This PDF file includes:

Figs. S1 to S34
Tables S1 to S4

Supplementary Figures

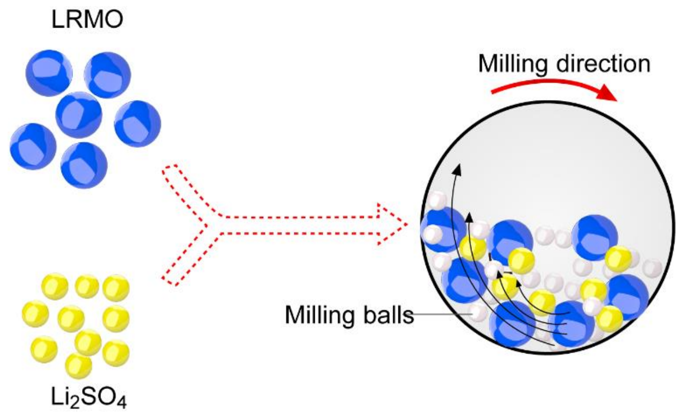

Fig. S1. Schematic illustration of the solid-state mechanochemistry process.

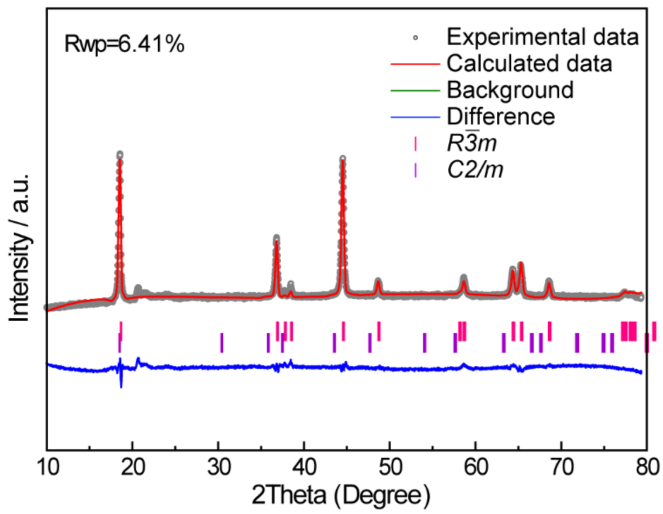

Fig. S2. Structure characterizations of pristine LRMO. XRD patterns with Rietveld fit for pristine LRMO; refinements are conducted on basis of the Li_2MnO_3 and $LiTMO_2$ biphasic model.

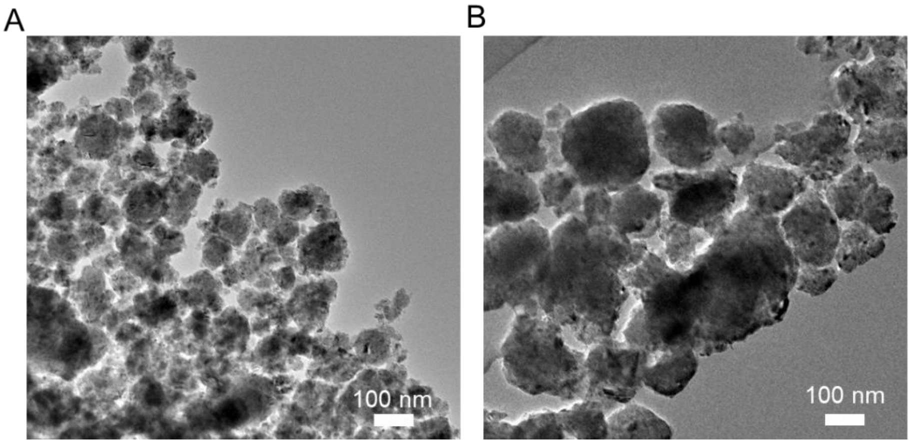

Fig. S3. Morphology characterizations of LRMO. (A, B) Low-magnification TEM images of B–LRMO and S–LRMO materials, respectively.

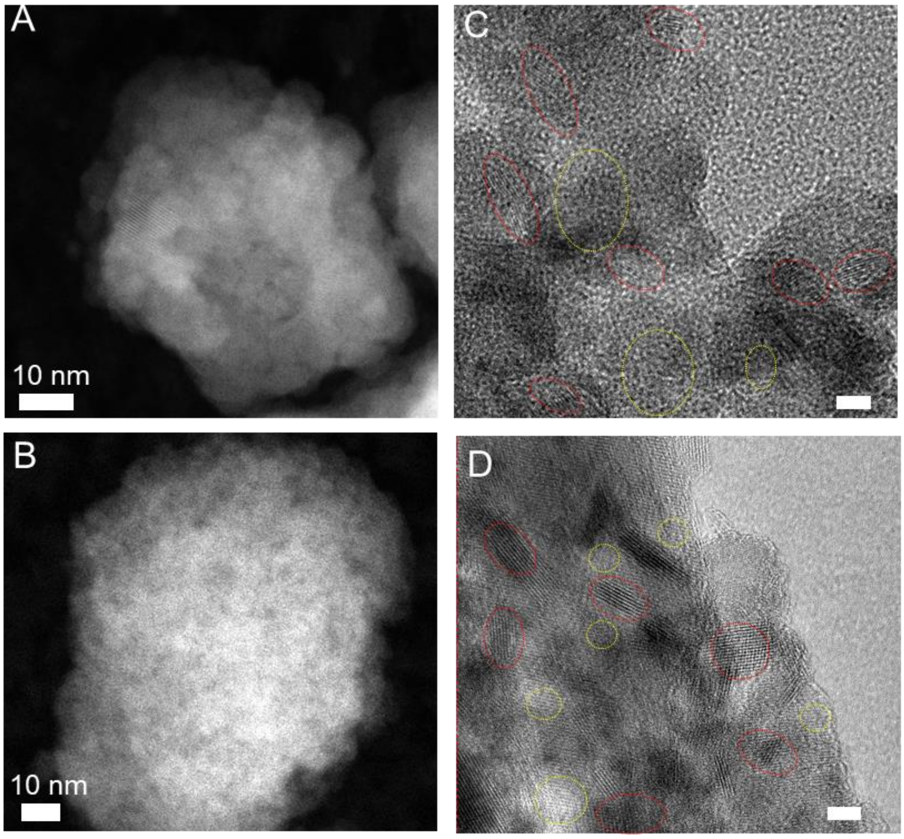

Fig. S4. Morphology and structure characterizations of LRMO. (A, B) The morphology of B–LRMO and S–LRMO nanoparticles. (C, D) The enlarged B–LRMO and S–LRMO nanoparticles with intergrown order-disorder domains; scale bar is 5 nm, suggesting the formation of intergrown domains of both S–LRMO and B–LRMO and a similar morphology of those after ball-milling treatments.

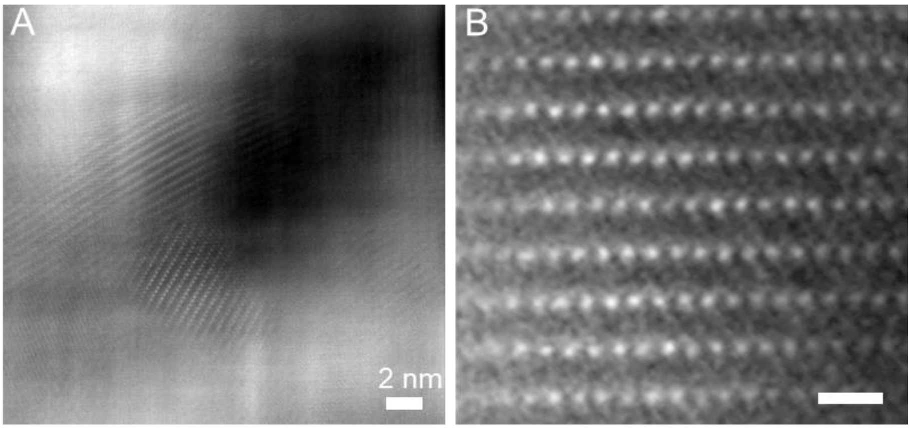

Fig. S5. Structure characterizations of B–LRMO at an atomic scale. (A) Representative HAADF-STEM image for the B–LRMO nanoparticle, showing the layered-rocksalt intergrown structure. (B) The layered structure in B–LRMO; scale bar is 0.5 nm.

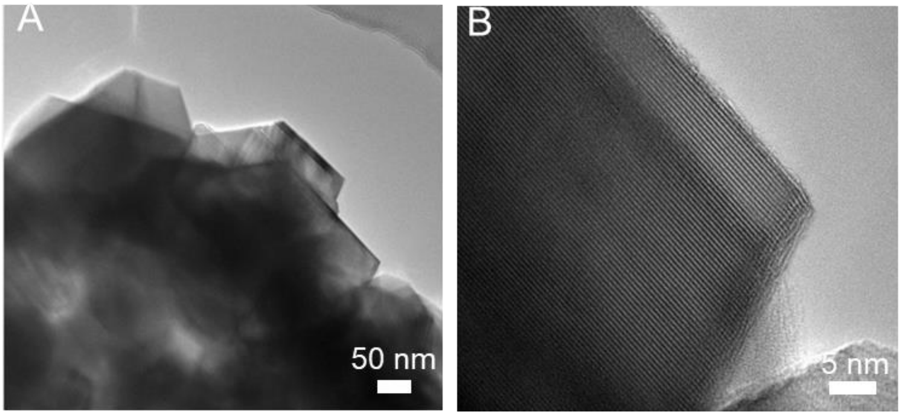

Fig. S6. Morphology and structure characterizations of pristine LRMO. (A, B) Representative TEM image for the pristine LRMO nanoparticle, showing the layered structure.

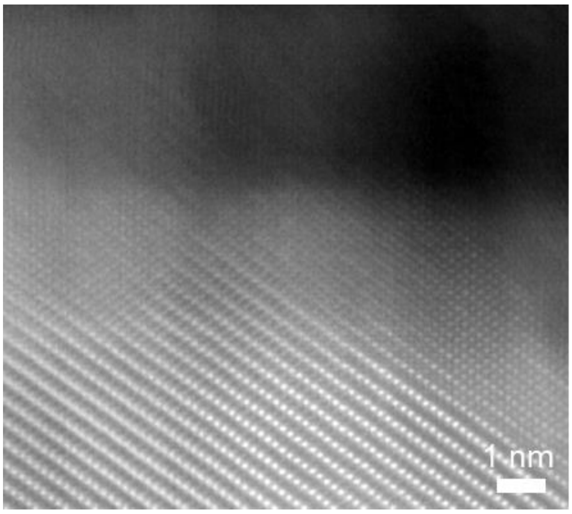

Fig. S7. **Structure characterization of interfaces in S–LRMO.** The enlarged interface in S–LRMO, showing gradual change from the layered structure to rocksalt structure.

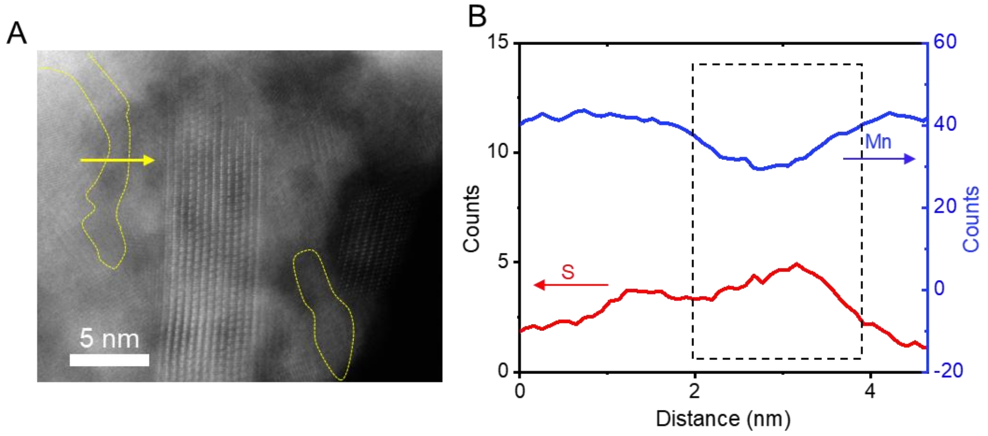

Fig. S8. Line-profile analysis at the atomic scale utilizing STEM/EDS. (A) HAADF images of the S–LRMO nanoparticle for line-scan analysis. (B) Distribution of components in the S–LRMO nanoparticle obtained by a line-scan analysis using EDS.

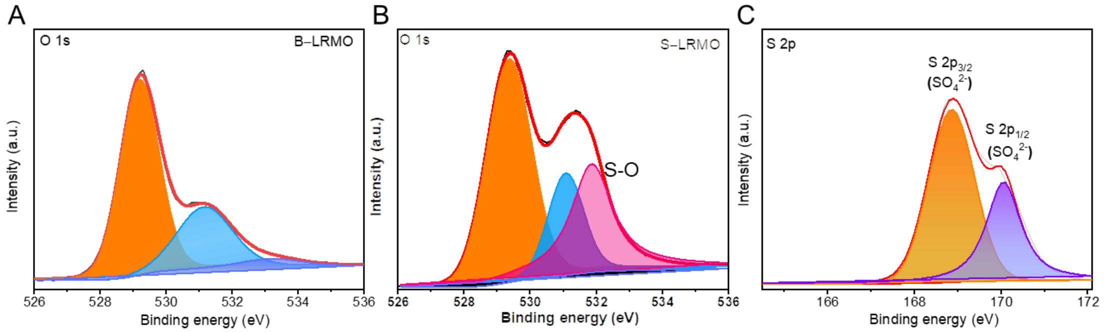

Fig. S9. The deconvoluted O 1s and S 2p XPS spectra. (A, B) O 1s XPS spectra of B–LRMO and S–LRMO materials. (C) S 2p XPS spectra of Li_2SO_4 after the ball-milling treatment.

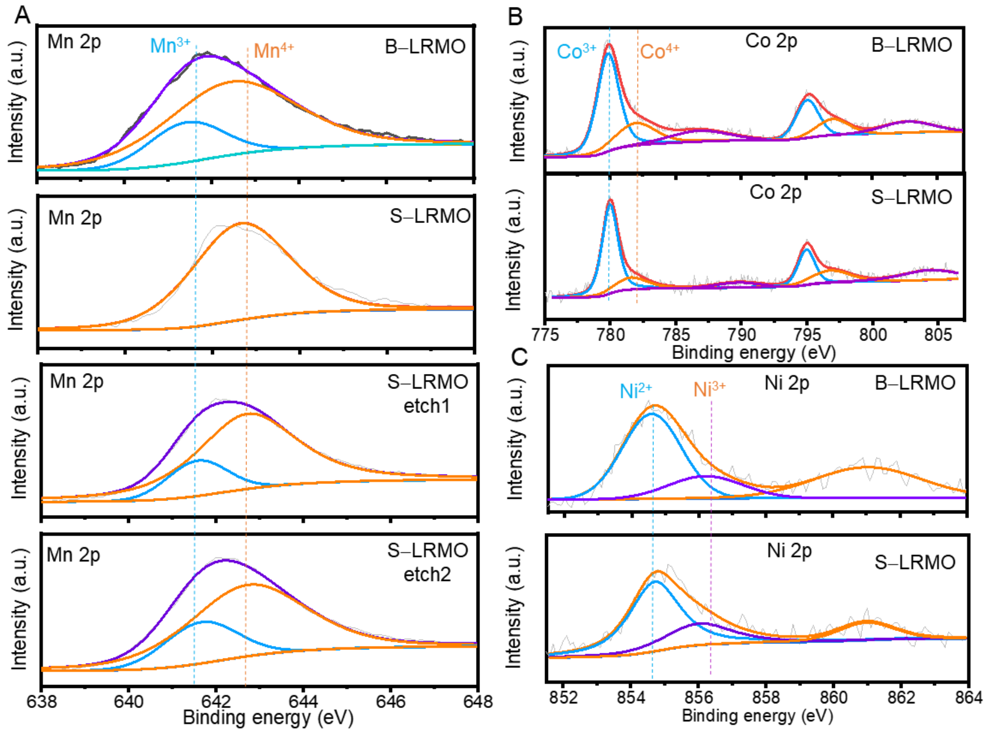

Fig. S10. The deconvoluted Mn 2p, Co 2p, and Ni 2p XPS spectra. (A) Mn 2p XPS spectra of B–LRMO and S–LRMO materials. Etch1 of S–LRMO represents the 5 nm etching depth and thus etch2 represents the10 nm etching depth. (B, C) Co 2p and Ni 2p XPS spectra of B–LRMO and S–LRMO, respectively.

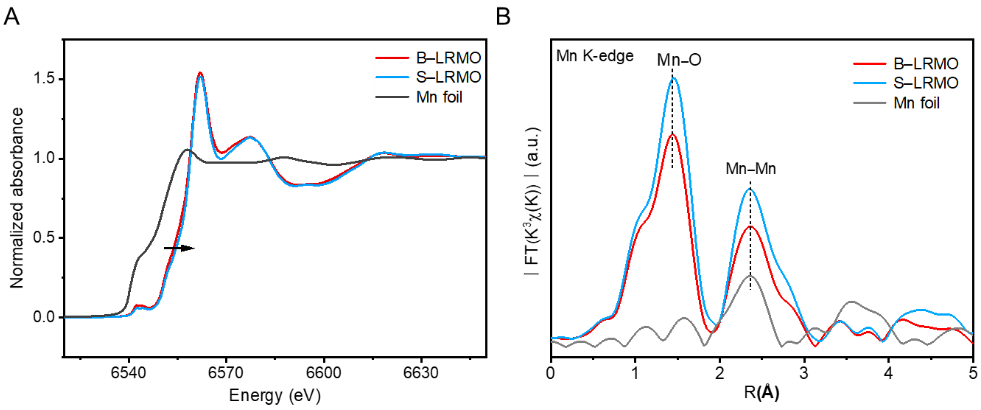

Fig. S11. Characterizations of the electron configurations and local structures. (A) Normalized Mn K-edge XANES spectra of B–LRMO and S–LRMO samples. (B) FT of k^3-weighted Mn K-edge EXAFS spectra for B–LRMO and S–LRMO samples.

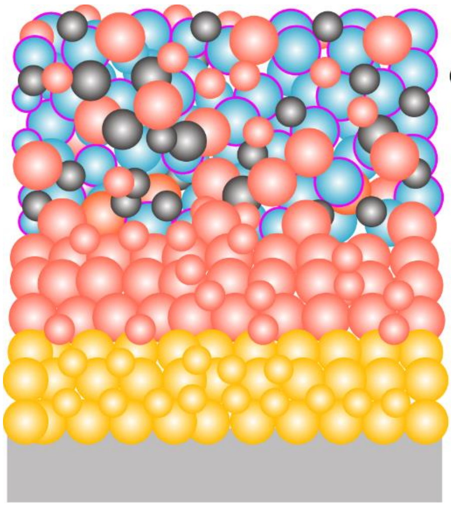

Fig. S12. The schematic illustration of the ASSB. The ASSB is based on the In/LiIn as the anode, Li_6PS_5Cl and $Li_3InCl_{4.8}F_{1.2}$ as the solid electrolyte, and the mixed LRMO cathode materials and $Li_3InCl_{4.8}F_{1.2}$ as the cathode composite.

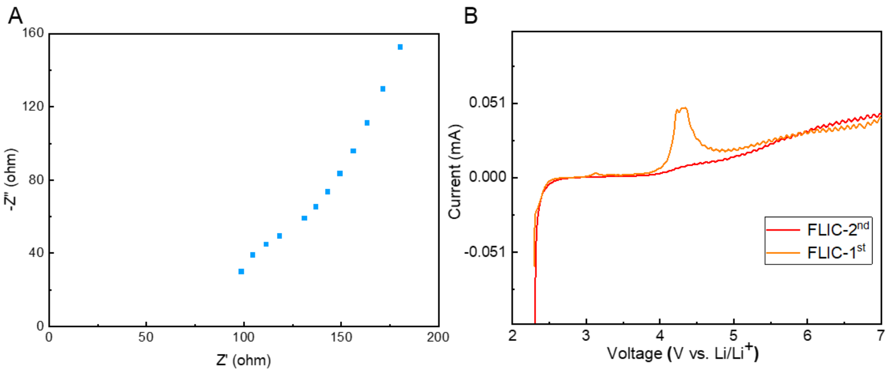

Fig. S13. Electrochemical measurements of the halide solid electrolytes. (A) Nyquist plots of $Li_3InCl_{4.8}F_{1.2}$; the thickness of tested $Li_3InCl_{4.8}F_{1.2}$ is ~400 μm. (B) First and second positive scans of the LICF from 2.3 V to 7.0 V (vs. Li/Li^+).

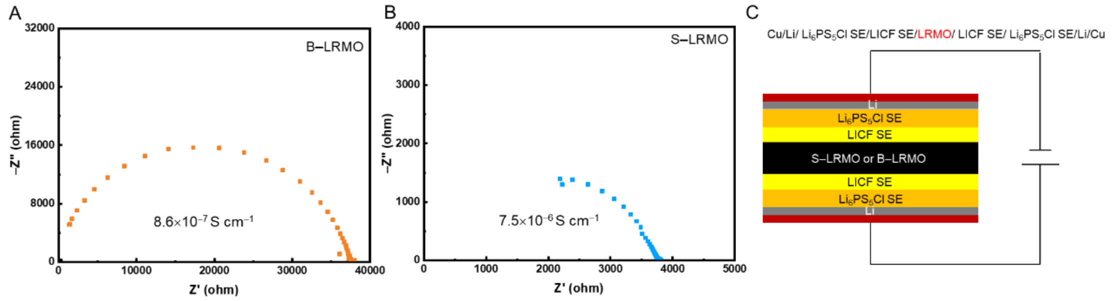

Fig. S14. Conductivity measurements of B–LRMO and S–LRMO materials. Nyquist plots of the symmetric cells to investigate the ionic conductivity of B–LRMO (A) and S–LRMO (B) materials using the Cu/Li/Li_6PS_5Cl SE/LICF SE/LRMO/ LICF SE/Li_6PS_5Cl SE/Li/Cu configuration (C) at 25°C.

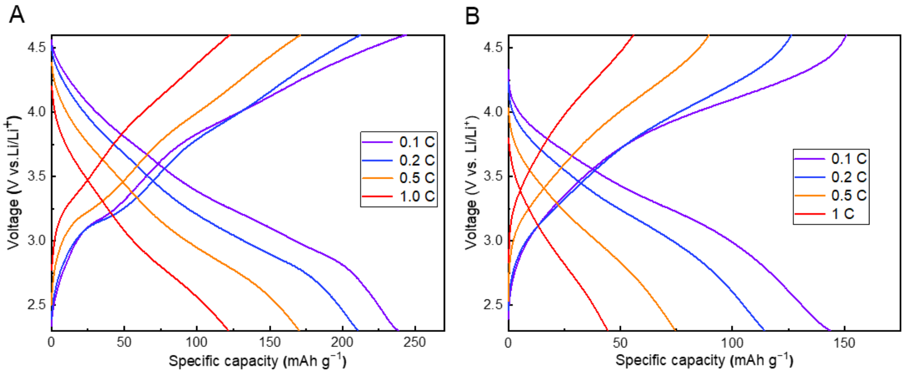

Fig. S15. The rate capability of S–LRMO and B–LRMO electrodes. Galvanostatic charge/discharge curves of (A) S–LRMO and (B) B–LRMO electrodes at the different current densities, respectively.

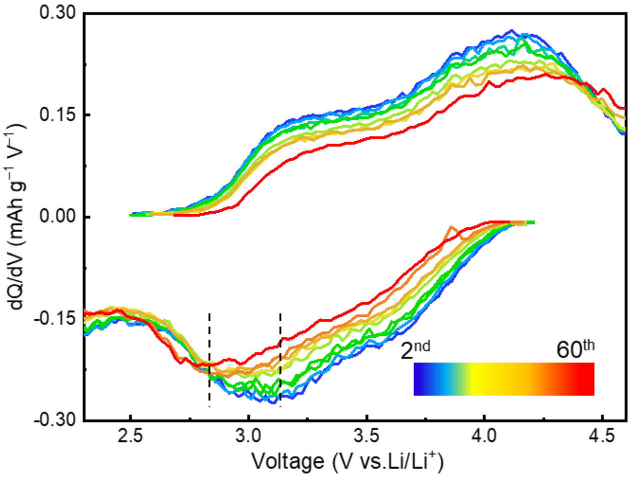

Fig. S16. Voltage degradation measurement for the B–LRMO ASSB. dQ/dV profiles of the charge/discharge processes of B–LRMO ASSBs collected from the 2nd to 60th cycle at 0.2 C.

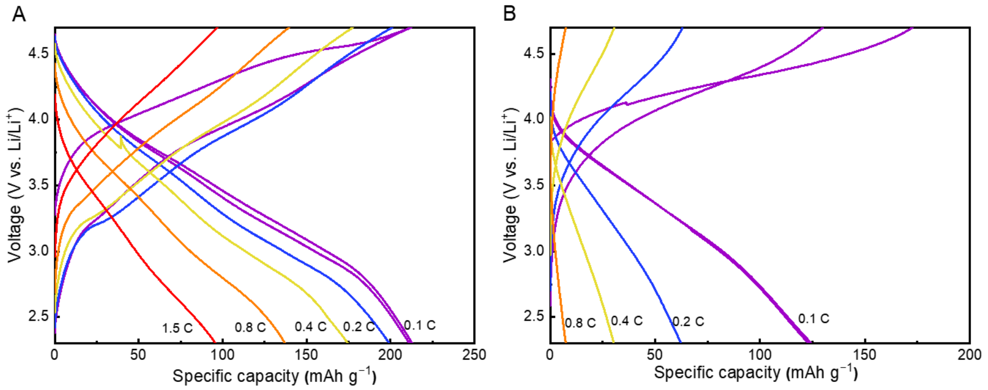

Fig. S17. Low-temperature performance of S–LRMO and B–LRMO at –10℃. Charge/discharge curves at different current densities for (A) S–LRMO and (B) B–LRMO ASSBs operated at –10℃.

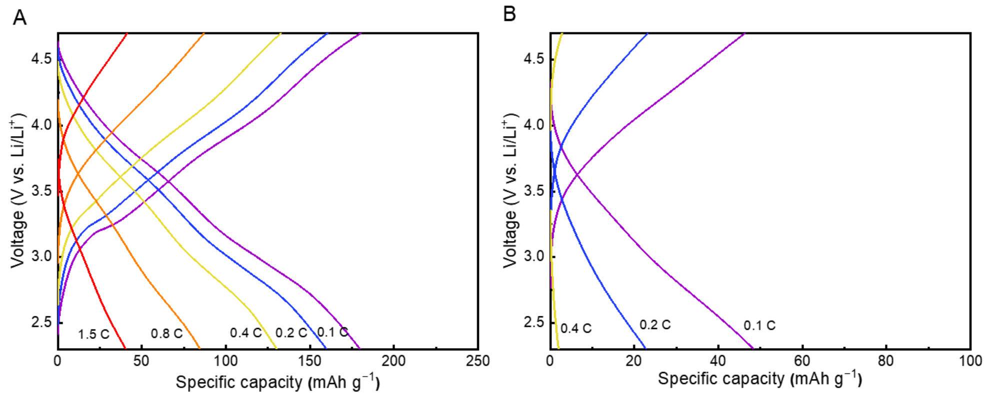

Fig. S18. Low-temperature performance of S–LRMO and B–LRMO at –20°C. Charge/discharge curves at different current densities for (A) S–LRMO and (B) B–LRMO ASSBs operated at –20°C.

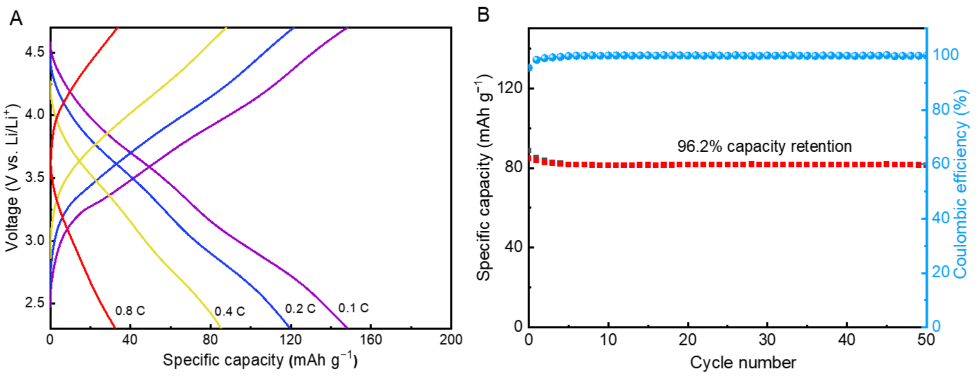

Fig. S19. Low-temperature performance of S–LRMO at –30℃. (A) Charge/discharge curves at different current densities for the S–LRMO ASSB operated at –30℃. (B) Cycling performance of S–LRMO ASSBs at 0.4 C operated at –30℃.

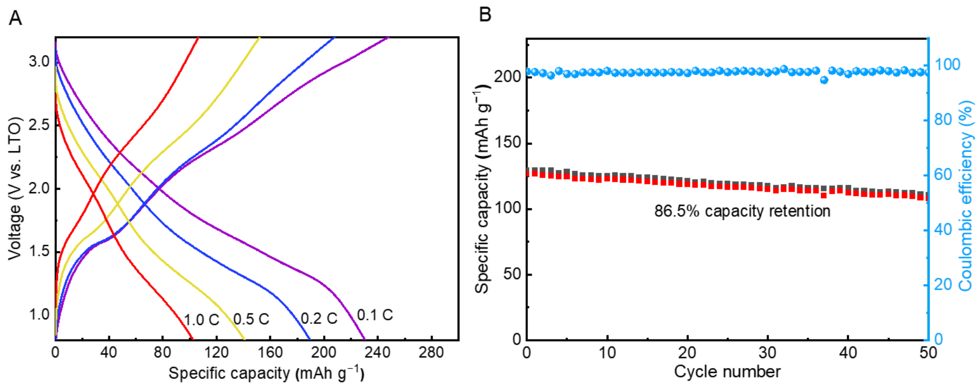

Fig. S20. The electrochemical performance of the S–LRMO/SE/LTO full cell. (A) charge/discharge profiles of S–LRMO electrode in S–LRMO/SE/LTO cell at different current densities. (B) Cycling performance of S–LRMO/SE/LTO full cell at 0.5 C.

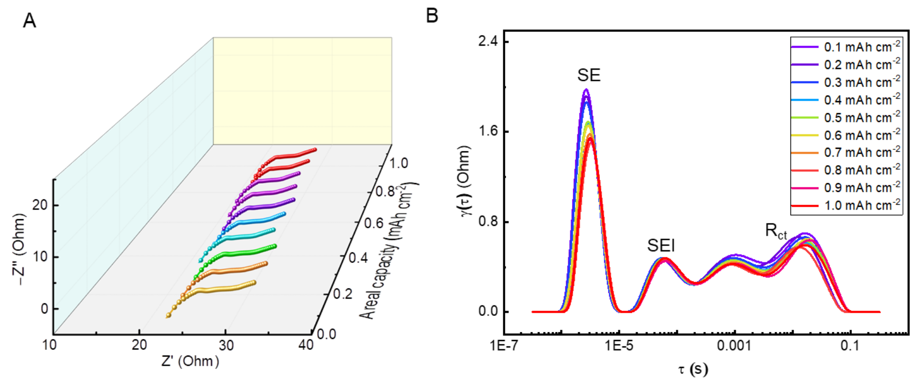

Fig. S21. Interfacial Li$^+$ transport evolution in the Li/Li symmetrical cell. (A) Interfacial impedance evolution of the Li/Li symmetrical cell charging about 1 mAh cm^{-2}. (B) The DRT profiles transformation of GEIS.

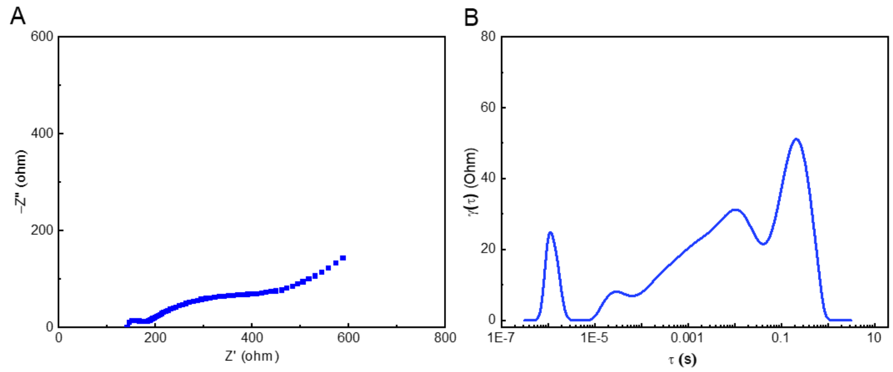

Fig. S22. Interfacial Li^+ transport in the S–LRMO (100% SOC)/S–LRMO (0% SOC) cell. (A) Interfacial impedance of the S–LRMO (100% SOC)/S–LRMO (0% SOC) cell and corresponding (B) DRT profiles transformation.

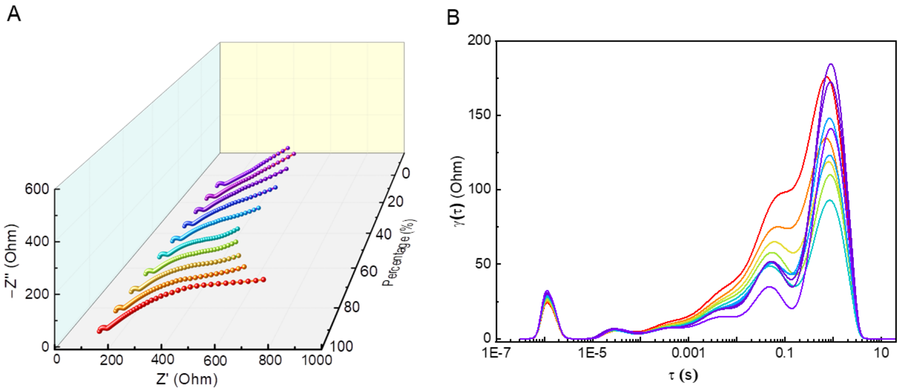

Fig. S23. Interfacial Li^+ transport evolution in the B–LRMO ASSB during discharging processes. (A) Interfacial impedance evolution of the B–LRMO ASSB; EIS spectra collected during initial discharging process. (B) The DRT profiles transformation of GEIS.

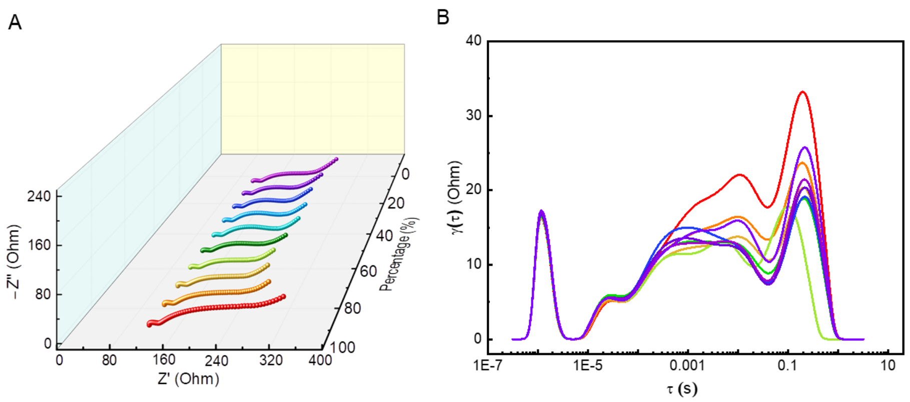

Fig. S24. Interfacial Li^+ transport evolution in the S–LRMO ASSB during discharging process. (A) Interfacial impedance evolution of the S–LRMO ASSB; EIS spectra collected during initial discharging process. (B) The DRT profiles transformation of GEIS.

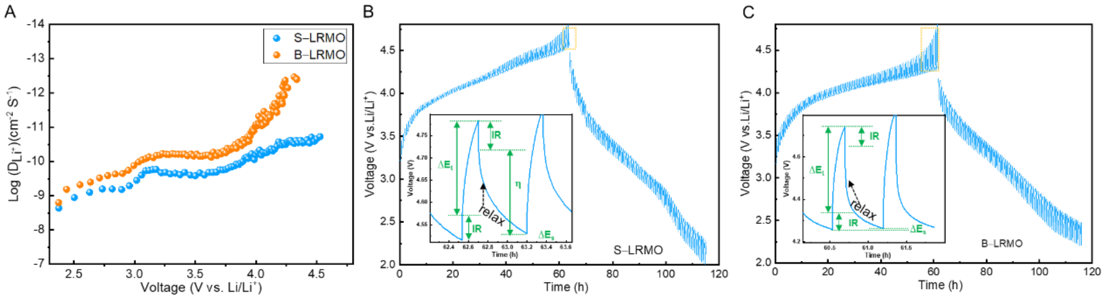

Fig. S25. The Li⁺ diffusion rates of S–LRMO and B–LRMO cathodes. (A) The D_{Li^+} of the S–LRMO and B–LRMO during the first charge process. GITT measurements at the initial cycle of (B) S–LRMO and (C) B–LRMO. Inserted is the enlarged voltage–time curve, where IR drops are marked, ΔE_τ represents the total battery voltage change, ΔE_s represents the changes in steady-state voltage.

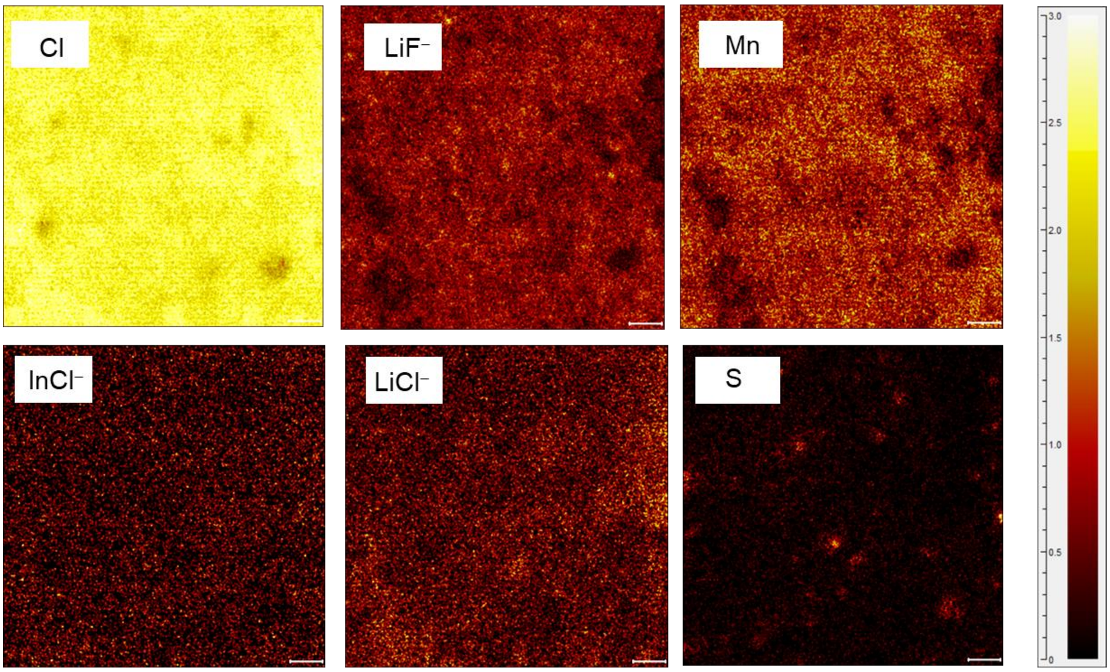

Fig. S26. TOF-SIMS analysis of B–LRMO cathode composites. The TOF-SIMS analysis results of B–LRMO cathode composites with cut-off potentials of 4.6 V vs. Li/Li^+ (1st); scale bar is 10 μm.

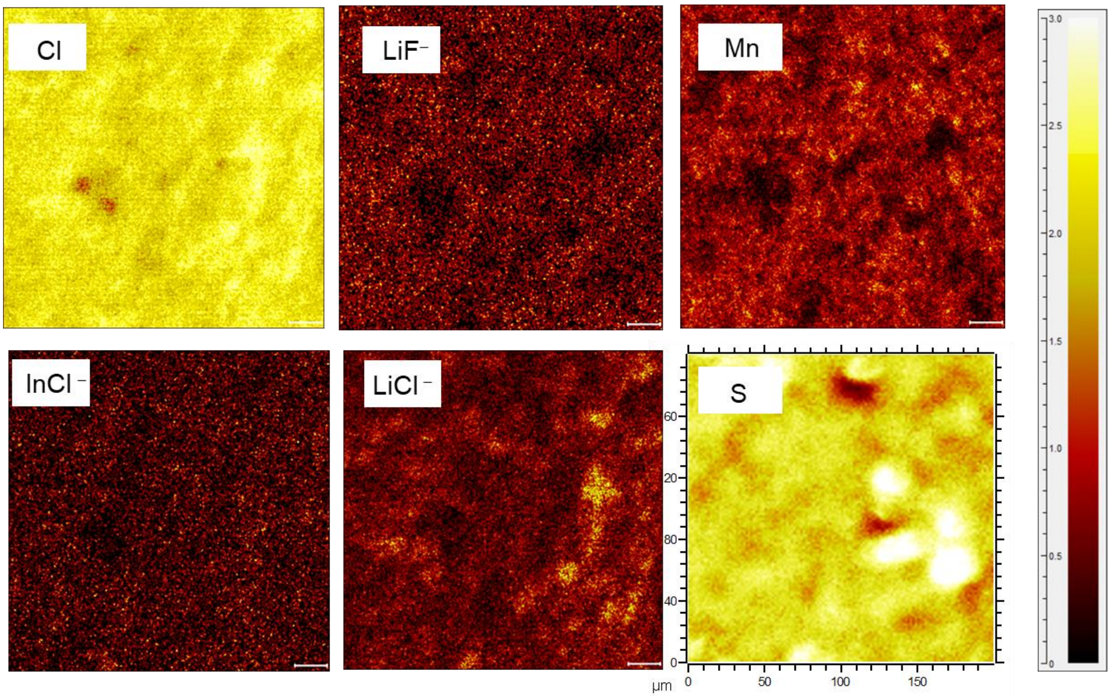

Fig. S27. TOF-SIMS analysis of S–LRMO cathode composites. The TOF-SIMS analysis results of S–LRMO cathode composites with cut-off potentials of 4.6 V vs. Li/Li^{+} (1st); scale bar is 10 μm.

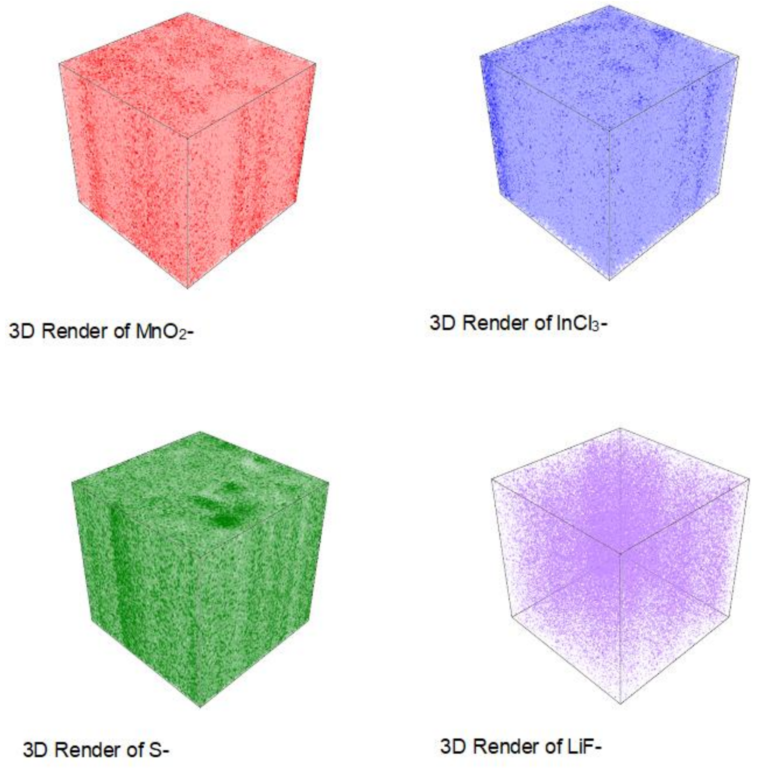

Fig. S28. 3D TOF-SIMS reconstruction of the cycled S–LRMO cathode. The fragment distributions of MnO_2^-, $InCl_3^-$, S^-, and LiF^- is shown, suggesting the uniform distribution of cathode materials and SE in the whole electrode.

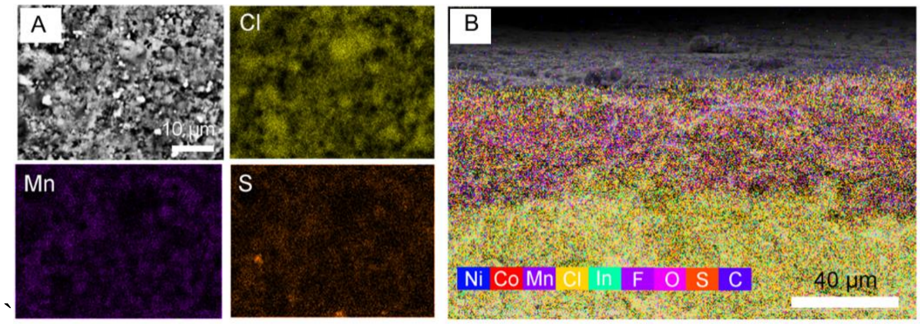

Fig. S29. EDS mappings analysis of the LRMO cathode. STEM image and corresponding EDS elemental mapping images of the cycled S–LRMO cathode (A) and the cycled S–LRMO│LICF interface (100 cycles) (B).

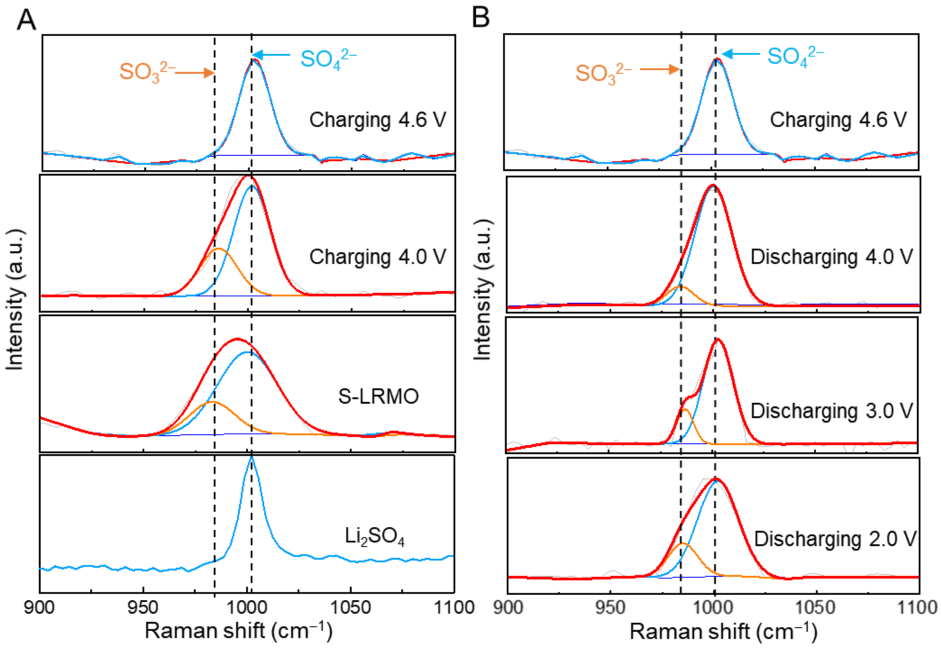

Fig. S30. ***Ex situ*** **Raman spectroscopy characterization of the S–LRMO cathode.** (A, B) Raman spectra for S–LRMO cathodes collected at the various charge and discharge states.

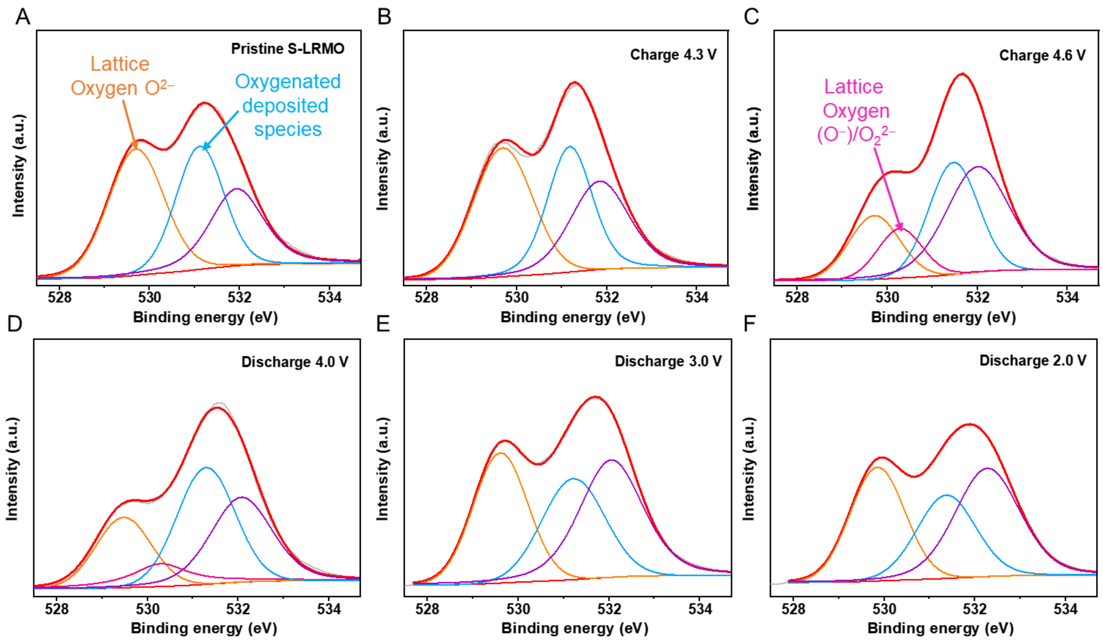

Fig. S31. Oxygen reactions for S–LRMO during cycling. (A–F) The O1s core spectra for S–LRMO electrode at the various charge and discharge states.

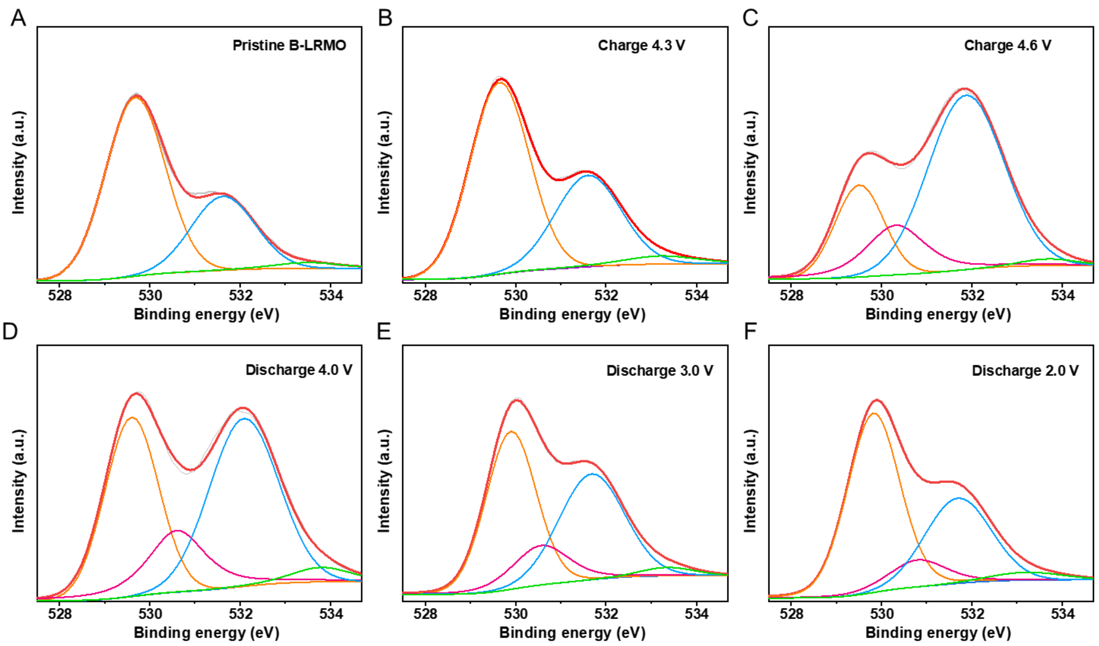

Fig. S32. Oxygen reactions for B–LRMO during cycling. (A–F) The O1s core spectra for B–LRMO electrode at the various charge and discharge states.

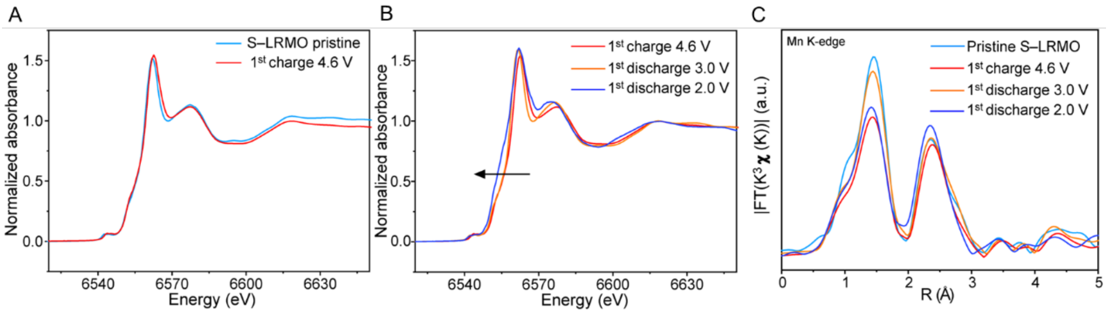

Fig. S33. Electronic structure of Mn probed by *ex situ* XAS. (A, B) Normalized Mn K-edge XANES spectra of S–LRMO collected at various charge and discharge states. (C) FT of k^3-weighted Mn K-edge EXAFS spectra for the S–LRMO collected at various charge and discharge states.

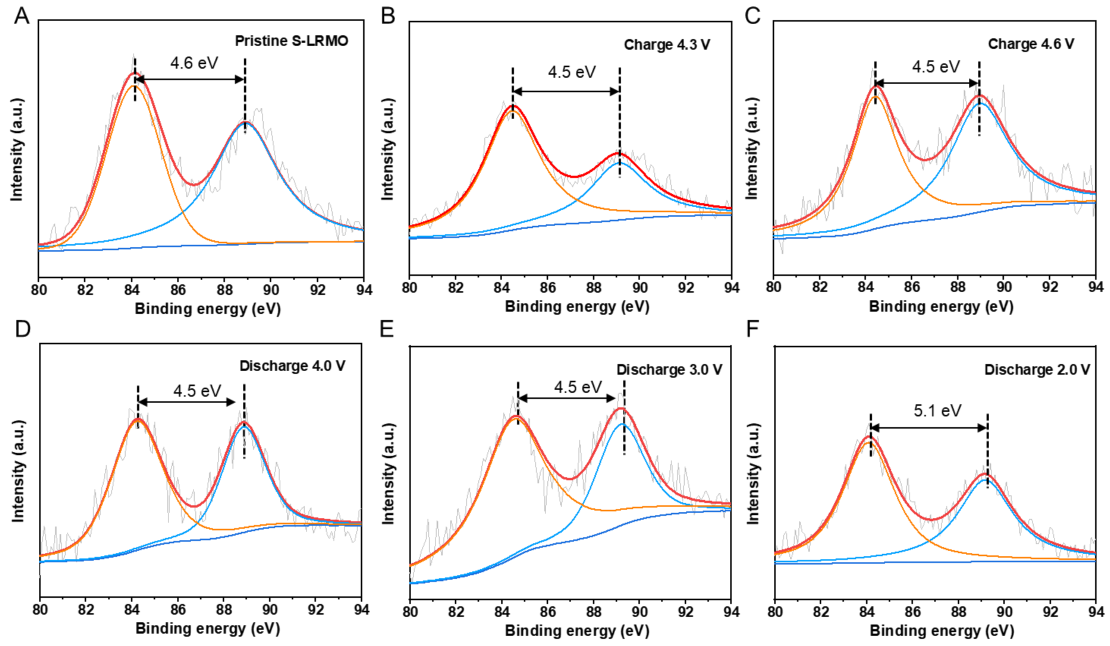

Fig. S34. Mn 3s XPS spectra of charged and discharged S–LRMO electrodes.

Table S1. The refined crystallographic parameters of the cathode materials by the XRD patterns.

Sample	a(Å)	c(Å)	O_z	V(Å^3)	R_{wp}(%)
pristine LRMO	2.85569(0)	14.27175(3)	0.24406	100.79(5)	6.41
B–LRMP	2.88905(2)	14.41214(5)	0.24916	104.17(1)	2.10
S–LRMO	2.85501(1)	14.25342(1)	0.24153	100.61(1)	2.33

Table S2. Atoms occupancy of pristine LRMO from Rietveld refinement by the XRD.

Atom	Site	*x*	*y*	*z*	Occ
Li	3a	0	0	0	0.983
Li1	3b	0	0	0.5	0.217
Ni	3b	0	0	0.5	0.113
Ni1	3a	0	0	0	0.017
Mn	3b	0	0	0.5	0.54
Co	3b	0	0	0.5	0.13
O	6c	0	0	0.24406	1

Table S3. Atoms occupancy of B–LRMO from Rietveld refinement by the XRD.

Atom	Site	*x*	*y*	*z*	Occ
Li	3a	0	0	0	0.885
Li1	3b	0	0	0.5	0.315
Ni	3b	0	0	0.5	0.015
Ni1	3a	0	0	0	0.115
Mn	3b	0	0	0.5	0.54
Co	3b	0	0	0.5	0.13
O	6c	0	0	0.24916	1

Table S4. Atoms occupancy of S–LRMO from Rietveld refinement by the XRD.

Atom	Site	*x*	*y*	*z*	Occ
Li	3a	0	0	0	0.906
Li1	3b	0	0	0.5	0.294
Ni	3b	0	0	0.5	0.036
Ni1	3a	0	0	0	0.094
Mn	3b	0	0	0.5	0.54
Co	3b	0	0	0.5	0.13
O	6c	0	0	0.24153	1
